# Untangle contributions of meteorology condition and human mobility to tropospheric NO_2_ in Chinese mainland during COVID-19 pandemic in early 2020

**DOI:** 10.1093/nsr/nwab061

**Published:** 2021-04-09

**Authors:** Zhang Yuxiang, Bo Haixu, Jiang Zhe, Wang Yu, Fu Yunfei, Cao Bingwei, Wang Xuewen, Chen Jiaqi, Li Rui

**Affiliations:** School of Earth and Space Sciences, University of Science and Technology of China, Hefei 230026, China; Comparative Planetary Excellence Innovation Center, Frontiers Science Center for Planetary Exploration and Emerging Technologies, Chinese Academy of Sciences, Hefei 230026, China; School of Earth and Space Sciences, University of Science and Technology of China, Hefei 230026, China; State Key Laboratory of Fire Science, University of Science and Technology of China, Hefei 230026, China; School of Earth and Space Sciences, University of Science and Technology of China, Hefei 230026, China; School of Earth and Space Sciences, University of Science and Technology of China, Hefei 230026, China; School of Earth and Space Sciences, University of Science and Technology of China, Hefei 230026, China; Jiangxi Ecological Environment Monitoring Center, Nanchang, 330000, China; Green Earth Science and Education Service, Slingerlands, NY, 12259, USA; School of Earth and Space Sciences, University of Science and Technology of China, Hefei 230026, China; School of Earth and Space Sciences, University of Science and Technology of China, Hefei 230026, China; Comparative Planetary Excellence Innovation Center, Frontiers Science Center for Planetary Exploration and Emerging Technologies, Chinese Academy of Sciences, Hefei 230026, China; State Key Laboratory of Fire Science, University of Science and Technology of China, Hefei 230026, China

**Keywords:** atmospheric nitrogen dioxide, anthropogenic emissions, meteorology conditions, human mobility, COVID-19 quarantine

## Abstract

In early 2020, unprecedented lockdowns and travel bans were implemented in Chinese mainland to stop the spread of novel coronavirus (COVID-19), which have led to large reduction of anthropogenic emissions. This provided a unique opportunity to isolate the effects from emission and meteorology on tropospheric nitrogen dioxide (NO_2_). Comparing the atmospheric NO_2_ in 2020 to 2017, we found the changes of emission have led to a -49.3 ± 23.5% reduction, which was ∼12% more than satellite observed reduction of -37.8 ± 16.3%. The discrepancy was mainly due to the changes of meteorology which have contributed to an 8.1 ± 14.2% increase of NO_2_. We also revealed that the emission induced reduction of NO_2_ has significantly negative correlations to human mobility, particularly that inside the city. The intra-city migration index derived from Baidu Location-Based-Service can explain 40.4%±17.7% variance of the emission induced reduction of NO_2_ in 29 megacities which each has a population of over 8 million in Chinese mainland.

## Teaser

The tangled effects from anthropogenic emission and meteorology condition on atmospheric NO2 in China was quantitatively isolated using satellite remote sensing, numerical modeling and traveling big data during the unprecedented COVID-19 related travel bans and lockdowns in early 2020.

ORIGINAL UNEDITED MANUSCRIPT

## Introduction

To curb the spread of the COVID-19, Chinese government implemented nation-wide strict control measures from the late January to March 2020 (1, 2). Lockdowns were executed in cities and provinces, leading to a gradually stopped inter-city and inter-province traffic (1). Inside cities and villages, strict self-quarantine have also been implemented. All people had to stay at home except for shopping living necessities or seeking medical treatment. Businesses and industries have suspended operation or reduced production largely. The intensive lockdown measures have led to dramatic decrease of human mobility (3, 4).

Nitrogen Dioxide ($NO_2$), as one of the most important air pollutants, is harmful to human respiratory system (5-7) and plays essential roles in the formation of acid rains, second order aerosols (8) and ozone (9-11). The dominant sources of tropospheric $NO_2$ over east China are anthropogenic combustions in winter, among which the contributions from power generation, industry and transportations are about 19%, 42% and 35%, respectively (12). The decrease of human mobility due to the lockdown measures is expected to have produced impacts on tropospheric $NO_2$ via affecting industry and transportation activities (13, 14). Recent studies have reported satellite observed large drop of column $NO_2$ density during this period due to the COVID-19 quarantine (1, 13, 15).

Besides anthropogenic emissions, tropospheric $NO_2$ concentrations are also strongly modulated by changes in meteorological conditions (16-19). Changes in wind speed, atmosphere stability (related to temperature and pressure etc.), solar radiation and humidity from day to day can quickly change the atmospheric $NO_2$ densities (16, 20). Temperature and humidity are crucial to the photochemical processes related to $NO_2$ (21). Higher temperature and higher humidity can reduce the lifetime of $NO_2$ and accelerate the conversion of $NO_2$ to secondary nitrate aerosols (17, 19) and thus lead to a negative correlation with atmospheric $NO_2$ concentration in most places (16, 19). Solar radiation is the key factor controlling the photodissociation rate of $NO_2$ ($NO_2 \rightarrow NO+O$), and can highly affect the lifetime of $NO_2$ (21, 22). This is strongly supported by the observation of increased $NO_2$ concentration during the solar eclipse (22). In general, surface $NO_2$ concentration is found to decrease with increasing solar radiation (16, 21). In addition, high wind speed and high planetary boundary layer height (PBLH) both favor the dispersion and dilution of air pollutants in the boundary layer of atmosphere (16, 17) and can reduce $NO_2$ concentration (21, 23, 24).

Anthropogenic emissions and meteorological conditions can both affect atmospheric $NO_2$ concentration, but their effects are often tangled. Although the reported literatures demonstrate the important influences of lockdowns on tropospheric $NO_2$ (13, 25), the respective contributions from anthropogenic and meteorological processes are not clear.

Chemical transport models are powerful to analyze the sources of atmospheric composition changes. However, the modelled results can be affected by potential uncertainties in the emission and chemistry processes. For example, *Liu et al.* (2018) shows that the modelled surface $NO_2$ over North China Plain is about 34% lower than surface measurement with GEOS-Chem model while is 26% higher than CMAQ model (26). Recent studies suggest the possibility to constrain the observation-based anthropogenic and meteorological influences with statistical models to avoid the effects of potential uncertainties in model simulations (27-29). During the early stage of COVID-19 pandemic, anthropogenic emissions in China were much lower than before (13, 15),

ORIGINAL UNEDITED MANUSCRIPT

while the changes of meteorology condition in 2020 were expected to be smaller than changes in emissions. It provides an ideal test-bed to study the separate impacts of emission and meteorological changes on atmospheric $NO_2$ with statistical models.

In this study, we investigated the effects of meteorology conditions and human mobility associated with COVID-19 quarantine on atmospheric $NO_2$ in China using a statistical model to represent the $NO_2$ (27). The human mobility strengths, including migration and intra-city flow were quantified using Baidu Migration data (3, 30). We focused on the month before (hereafter Month-01) and after (hereafter Month-02) the Chinese Spring Festival in 2017, 2018, 2019 and 2020 to take the holiday effect on human mobility into account.

**Results**

**A statistical model of troposphere $NO_2$**

According to the annual Report on the State of the Environment in China from 2015 to 2019 (http://english.mee.gov.cn/Resources/Reports/soe/), the mean $NO_2$ concentrations of the cities in China were relatively stable from 2017 to 2019. Before that, anthropogenic NOx emissions (normalized in 2010) were reduced by about 21% in 2012-2015 (7%/year) and about 6% in 2015-2017 (3%/year) (12). The dramatic declines of anthropogenic NOx emissions in 2012-2015 as well as the subsequent slowdown of emission reductions were mainly driven by the installation of selective catalytic reduction (SCR) systems in utilities for coal-fired power plants(12).

Recent studies have revealed that the satellite observed column $NO_2$ density in China in 2020 Month-02 was much lower than that in 2019 (15) and concluded that this drop was attributed to the COVID-19 related city lockdowns and travel bans (1). However, it must be recognized that the atmospheric $NO_2$ concentration is also highly affected by meteorology conditions (15).

Assuming the real measurements of Atmospheric $NO_2$ (in logarithm) can be separated into two parts, $NO_2$ contributed by emission $F_{Emis}(x,t)$ and by meteorology conditions $G_{Mete}(x,t)$, we have the following function associated with geolocation (x) and time (t) (27):

$$\log NO_2(x,t)^{OBS} = F_{Emis}(x,t)^{OBS} + G_{Mete}(x,t)^{OBS} \quad (1)$$

A statistical model of $\log NO_2(x,t)^{MOD}$ was established on two assumptions. (1) The regression model was trained in Month-01 and Month-02 in 2018 and 2019. Considering the fairly stable $NO_x$ emission in China in the recent years (12), $F_{Emis}(x,t)$ based on 2018 and 2019 should provide a good approximation for the anthropogenic influences for 2017 and 2020. We thus, assumed the temporal variations of $F_{Emis}(x,t)$ is negligible at each grid of 0.5×0.5 degree. (2) The temporal and spatial variations of $G_{Mete}(x,t)$ can be modelled using a simplified linear function of five key meteorology parameters as described in **Data and Methods**. We found these two assumptions resulted in good agreements between the observed and modelled tropospheric $NO_2$, based on self-consistency check (using training data) and independent check (using independent data). Therefore, we have:

$$\log NO_2(x,t)^{MOD} = F_{Emis}(x)^{MOD} + G_{Mete}(x,t)^{MOD} \quad (2)$$

It should be noted that the modelled emission term $F_{Emis}(x)^{MOD}$ in Eq.2 is only a function

ORIGINAL UNEDITED MANUSCRIPT

of geolocation. In other words, its value keeps constant at given 0.5×0.5 degree box based on statistical regression. The detailed regression procedures and sensitivity tests are described in **Data and Methods**. The difference between satellite observations and modelling results can be expressed as:

$$\mathrm{Log}\left[\frac{NO_2(x,t)^{MOD}}{NO_2(x,t)^{OBS}}\right] = [F_{Emis}(x,t)^{MOD} - F_{Emis}(x)^{OBS}] + \Delta \quad (3)$$

where the first term at the right hand of Eq.3 represents the error introduced by ignoring the temporal variations of emission. The second term $\Delta$ represents the modelling error of $G_{Mete}(x,t)$.

The performance of the model was analyzed using independent (from establishing the model) observations in 2017. The model successfully predicted the monthly mean atmospheric $NO_2$ in 2017 Month-02 (**Figure 1**) with negligible bias in most areas in China. Even in the heaviest polluted areas in central and eastern China with $NO_2$ over $10\times10^{15}$ molec/cm$^2$, the mean bias is only 4.3% comparing to satellite observations. On the other hand, the spatial correlation coefficients between model prediction and satellite $NO_2$ are as high as 0.97 ($p<0.001$). Similar results for 2018 and 2019 can be seen in **Figure S4**.

The model also works well at predicting daily $NO_2$. At the scales of provinces (**Figure S5**) and cities (**Figure S6**), statistically significant positive correlation coefficients between daily mean $NO_2$ predicted by the model and the real satellite measurements can be found in most areas in the mainland of China in Month-01 and Month-02 in 2017, 2018 and 2019.

The above results demonstrated that the contribution of emission to the atmospheric $NO_2$, in the same month of the adjacent years, can be estimated as a time-independent geolocation-based function. The spatial and temporal variations of meteorology effects can be modelled using the linear function of five selected key parameters. The modelling error is generally less than 5% based on validations in 2017. The underneath reason for the emission situation in 2017 was similar to that in 2018/2019, and the regression model captured the quantitative dependence of atmospheric $NO_2$ on meteorology conditions. Consequently, if the anthropogenic emissions in 2020 was similar to that in 2018/2019 (i.e., without the effects from COVID-19 quarantine), the model was expected to provide good prediction for tropospheric $NO_2$ in 2020.

It should be noted that changes of column atmospheric $NO_2$ is not linearly associated with the emission due to the nonlinear effect from atmospheric chemistry. Based on the results from GEOS-Chem chemical transport model study (**Figure S17**), we found broadly linear response of modelled tropospheric $NO_2$ columns to changes in anthropogenic NOx and VOCs emissions, e.g., 50% reduction of anthropogenic emissions results in about 45% reduction of tropospheric $NO_2$ columns. The model simulations suggest that the influence from nonlinear processes is small (about 5%).

**Quarantine-induced reduction in troposphere $NO_2$**

In 2020, although the $NO_2$ variations related to meteorology conditions could still be modelled with good accuracy, the emissions of $NO_2$ were significantly reduced due to the city lockdowns

ORIGINAL UNEDITED MANUSCRIPT

and travel bans. Therefore, the foundation of the statistical model describing the contribution of emission collapsed. The term of $F_{Emis}(x,t)^{OBS} - F_{Emis}(x)^{MOD}$ in Eq.3 in 2020 became much larger than that in 2017. The model overestimated monthly mean $NO_2$ by 6 - 9×$10^{15}$molec/cm$^2$ in the heavily polluted areas in China (**Figure 1d**). Similar overestimation also could be seen from the time series of daily mean $NO_2$ in most polluted cities and provinces (**Figure S7-S10**) such as Tianjin, Shanghai, Shandong, Jiangsu, and Beijing.

If we compared the satellite observation of atmospheric $NO_2$ in 2020 to that in 2017, we could decompose the difference into three isolated terms (31):

$$\begin{aligned} & NO_2(x,2020)^{OBS} - NO_2(x,2017)^{OBS} \\ & = [NO_2(x,2020)^{OBS} - NO_2(x,2020)^{MOD}] \\ & +[NO_2(x,2020)^{MOD} - NO_2(x,2017)^{MOD}] \\ & +[NO_2(x,2017)^{MOD} - NO_2(x,2017)^{OBS}] \end{aligned} \tag{4}$$

where the first term represents the emission-induced reductions in 2020; the second represents the meteorology induced variations; and the third represents the modelling error. Using real satellite observations and modelling results in 2020 and 2017, the map of the contributions (unit: %) from emission and meteorology (i.e., the above three terms) to the reduction of $NO_2$ is shown in **Figure 2**. In most China's cities with monthly mean $NO_2$ over 3×$10^{15}$molec/cm$^2$, we found that emission-induced reductions in 2020 (**Figure 2b**) was larger than the satellite observed reduction (**Figure 2a**), because the meteorology in 2020 led a net increase of $NO_2$ comparing to 2017 (**Figure 2c**).

Overall, the OMI observed troposphere $NO_2$ decreased by -37.8±16.3% in 2020 comparing to that in 2017. Using the model mentioned above, we estimated that if the weather conditions in 2020 were the same as in 2017, the $NO_2$ columns should decrease by -49.3±23.5% because of the reduced anthropogenic emission, which means the emission-induced reduction of $NO_2$ due to the COVID-19 quarantine was actually higher than OMI actual observations. The meteorological conditions in 2020 did not favor the dilution and ventilation of air pollutants, and thus have led to an increase of $NO_2$ of 8.1±14.2%. Meanwhile, the modelling error of the above estimation is only -3.32±17.8% which is significantly smaller than the other two terms.

The statistic model results are consistent with GEOS-Chem model simulations. As shown in **Figure 2e**, there are good agreements in the derived impacts of meteorological variability. Both GEOS-Chem based and statistic based results show positive contributions from meteorological variability in northern China, and neutral and weakly negative contributions in central and southern China. Quantitatively, the weather effects from GEOS-Chem and statistic based analysis are 17.42±22.90% versus 20.38±15.55% over the selected North China Plain area, -2.32±7.67% versus 2.12±5.18% in Anhui, and -3.27±18.41% versus -2.78±7.99% in Guangdong.

The results are also consistent with other studies in the literature (14, 32-34). Zhang et al. (2020) estimated the daily NOx emission in 2020 by combining TROPOMI $NO_2$ observation with WRF-GEOSChem simulations, and reported a 50% decrease of emission after the COVID-19 lockdown (14). Marlier et al. (2020) observed a 49% decline of $NO_2$ after Lunar New Year, and found the weather conditions weakened the emission reduction (33). Zhao et al. (2020) applied the

ORIGINAL UNEDITED MANUSCRIPT

emission inventory of 2017 to WRF-CMAQ, and concluded that the meteorological condition in 2020 elevated the $NO_2$ concentration in over half of the cities in China (34). Wang et al. (2020) found the reduction of PM2.5 simulated by WRF-CMAQ is smaller than the reduction of precursor emissions, also indicating the unfavorable meteorology (lower PBLH, WS and higher RH) for the dilution of the pollutants (35).

Therefore, the COVID-19 quarantine actually has caused a reduction of $NO_2$ larger than we saw from the satellite observation (i.e. a direct comparison of 2020 to 2017), but the weather effect has cancelled out some of the emission effect. The modelling error is significantly smaller than each of the two effects, particularly the mean value. This is the first time to isolate contributions of emission and weather to the satellite observed reduction of $NO_2$ in early 2020. Similar analyses using situations in 2018 and 2019 were given in **Supplementary Material** (**Figure S11-S12**). Overall, when comparing 2020 to 2018 and 2019, the emission induced reduction of $NO_2$ also was significantly larger than the satellite observed reduction due to the meteorology contribution. This confirmed the conclusion derived from the 2020-2017 comparison.

**Human mobility and $NO_2$**

Based on Eq.3, the difference between satellite observations and model prediction in 2020, i.e. $\mathrm{logrNO_2}(2020) = \mathrm{logNO_2}(x, 2020)^{MOD} - \mathrm{logNO_2}(x, 2020)^{OBS}$ mainly represents the emission related reduction of $NO_2$ (hereafter $\mathrm{logrNO_2}$), which can be attributed to multiple factors including the prohibition of human mobility implemented by the government, the close of businesses consuming fusel fuel such as restaurants, hotels etc., the reduction of industrial production due to weakened domestic and international trades etc. It is hard to make a thorough survey to measure all of those factors in the current situation when COVID-19 remains a serious threaten to human health.

Fortunately, satellite location-based services (LBS) describing the human mobility (36) are useful proxies of anthropogenic emissions. To what extent and by which the emission related reduction of $NO_2$ in 2020 can be explained by these new type of LBS data? In order to quantitatively understand the roles of city lockdowns and travel bans played in reducing the air pollutants in China, we investigated the correlations between the emission related reduction of $NO_2$ and Baidu migration data (**Data and Methods**) of three indices representing the relative population flow moving in (I-index), moving out (E-index) and moving inside the city (C-index). The spatial pattern of the indices in the mainland of China in Month-01 and Month-02 in 2019 and 2020 are shown in **Figure S13**. Generally, values are higher in eastern China than those in western China. The spatial patterns and inhomogeneities are associated with population density and economy activities. For example, those megacities such as Beijing, Shanghai, and Guangzhou showed large values of all indices because of their dense population, large number of migrant workers and prosperous economy.

As shown in **Figure 3**, since people started to go home for family reunion in Month-01 2020, the immigration index (I-index, red curves) and emigration index (E-index, dash red line) in megacities with population over 8 million rapidly increased and peaked around Jan 23$^{rd}$ 2020 when Wuhan, Hubei was locked down due to the COVID-19 outbreak. After that, both I-index and

ORIGINAL UNEDITED MANUSCRIPT

E-index decreased sharply and remained in very low values during Month-02 2020 (4). In Month-01shopping and visiting inside cities also increased for preparing the coming Spring Festival, and the intra-city index of C-index (blue curves) also was high before Jan 23rd 2020. For the same reason, the C-index dropped after the lockdowns. The "Spring Festival Effect" also was shown in 2019 except that the C-index picked up quickly several days after the Spring Festival.

Meanwhile, the emission related change of logr$NO_2$ (vertical bars in **Figure 3**) in 2020 just oscillated around zero in each day before Jan 23rd. After that, it held positive values and was significantly larger than the standard variations. In the contrast, in 2019, the difference between satellite observation and model prediction of $NO_2$, i.e. logr$NO_2$, maintained small values during the whole period of Month-01 and Month-02, and the temporal variations were always within the range of standard deviation. The comparison between 2020 and 2019 confirmed that the emission changes in 2020 induced large reduction of $NO_2$.

To investigate the quantitative relationship between logr$NO_2$ and the human mobility indices, we presented the scatter plots of daily logr$NO_2$ to daily indices in 2019 and 2020 in cities each with population of over 8 million in the lower panel in **Figure 3**. In 2019, there were almost no statistically significant correlations between logr$NO_2$ to all types of migration indices because the emission induced variations of $NO_2$ was very small. While in 2020, daily logr$NO_2$ in individual city or averaged in all cities both negatively correlated with those migration indices with $P<0.001$. The intra-city migration index C-index showed the strongest correlation and could explain 22.1% (i.e. the $R^2$ at individual city level) to 60.8% (the $R^2$ averaged in all megacities) variance of the logr$NO_2$. The immigration index I-index could explain 13.0 to 36.0%; the emigration index E-index could explain 12.3 to 27.0%.

A list of the explained variance of logr$NO_2$ by migration indices (from high to low) of all 29 megacities were given in **Table 1**. In some of the cities in Southern China such as Guangzhou and Dongguan, C-index could explain as high as ~70% variance. In Mid and Eastern China, cities like Suzhou, Heze, and Xuzhou also showed explained variance of over 60% by the C-index. This indicates the human mobility inside the city is more important to affect the emission of $NO_2$ than the population flow toward (I-index) or out (E-index) of the city.

Not just in those megacities, negative correlations between logr$NO_2$ and migration indices were also seen nation widely in the mainland of China in 2020. In **Figure 4**, among cities with mean $NO_2$ over $3\times10^{15}$ molec/cm$^2$, the logr$NO_2$ showed significantly negative correlations with I-index in 116 cities and with E-index in 110 cities, with explained variance of $0.203\pm0.120$ and $0.208\pm0.124$, respectively. For intra-city index, C-index, 156 cities showed significantly negative correlations with explained variance of $0.279\pm0.159$. No city showed positive correlation with logr$NO_2$ for the C-index. The results indicated that these LBS based migration indices, particularly the intra-city index (C-index), to a large extent, provided reasonable explanation for the temporal variations of emission induced reduction of $NO_2$ in large areas of China in early 2020.

ORIGINAL UNEDITED MANUSCRIPT

## Discussion and Conclusion

China has paid great efforts with huge economy losses to stop the spread of COVID-19 in 2020 (1). The unprecedented lockdowns and travel bans have led to large reduction of anthropogenic emission of air pollutions (13). There are several studies reported the reduction of air pollutants in Early 2020 in China and attributed that to the effect of COVID-19 quarantine without considering the effect of meteorology. In this study, we took the opportunity of this unintentionally conducted circumstance to investigate the isolated effects from emission and meteorology condition on atmospheric $NO_2$, and the quantitative relationship between the reductions of $NO_2$ to human mobility using state-of-the-art satellite remote sensing product and location-service-based big data. We established a statistical model representing the column density of $NO_2$ as a function of only five key meteorology parameters with the assumption that emission was constant. Comparing to satellite observations in early 2017, the model predicted monthly mean $NO_2$ was only biased by 4.3% in the heaviest polluted areas in central and eastern China and showed spatial correlation coefficient of 0.97 ($P<0.001$).

By using the statistical model, it was found that the travel bans and lockdowns of China in 2020 have resulted in the decrease of observed $NO_2$. Meanwhile, the changes of meteorological conditions, such as lower PBLH, lower solar radiation etc., have led to an increase of atmospheric $NO_2$ (**Figure 5**). As shown in **Figure 5**, comparing to 2017, the anthropogenic emission changes in early 2020 have led to a $-49.3\pm23.5\%$ reduction of atmospheric $NO_2$, and meanwhile, the changes of meteorology conditions have led to an $8.1\pm14.2\%$ increase. Consequently, the net reduction of $NO_2$ in 2020 comparing to that in 2017 was brought down to $-37.8\pm16.3\%$. And that the modelling error was $-3.3\pm17.8\%$. It is the first time to reveal that the COVID-19 quarantine caused reduction of atmospheric $NO_2$ actually was larger than what we saw from the satellite observations. In addition, the emission induced reduction of $NO_2$ shows statistically significant correlations to human mobility. Quantitatively, the migration index representing the movement inside the city has the highest explained variance among all indices: it can explain $40.4\%\pm17.7\%$ variance on average in 29 megacities each with population of over 8 million in the mainland of China. This study established a method to untangle the contribution of emission and meteorology condition to the reduction of atmosphere $NO_2$ in early 2020, and quantitatively assessed the effect of the city lockdowns and travel bans implemented by China's government in response to the COVID-19 on the tropospheric $NO_2$ reduction. This analysis may shed a light onto the parameterization of $NO_2$ emission related to human mobility, as well as the understanding of the effect of transportation on atmosphere $NO_2$. The indices based on the Baidu Big Data are able to provide the daily information of human activities, and thus, can predict the change of $NO_2$. In the future, the data can be used to modify the emission model and make the emission estimation more accurate (37, 38).

In this study, we established a simplified model (27) to describe the dependence of atmospheric $NO_2$ on emission and meteorology. Comparing to those physical and chemical transfer models, which require large source of computing time (26) and are affected by uncertainties in modelled physical or chemical processes, the approach shown in this work is fast

ORIGINAL UNEDITED MANUSCRIPT

and accurate with relative error less than 5% even in the heaviest polluted area in China, provided the emission does not change significantly. It demonstrates the reliability of statistic approaches to predict tropospheric $NO_2$ changes.

The impacts of COVID-19 controls on tropospheric $NO_2$ have attracted substantial attentions. However, it remains a challenge to interpret the observed tropospheric $NO_2$ changes, particularly when considering the large influence of meteorological variabilities. This work shows that meteorological variabilities have resulted in about 20% enhancement of tropospheric $NO_2$ column over northern China in 2020. It is an important advance to improve our understanding about the responses of tropospheric $NO_2$ to perturbations in anthropogenic activity changes.

We suggest more efforts to develop novel statistic-based approaches as important supplements to the chemical transport models, particularly to understand the sensitivity of $NO_2$ to various meteorological variables in order to provide more accurate predictions. We admit that the uncertainty in statistic model deserves further studies, in particular its dependence on the background of $NO_2$ concentration, as well as the sensitivity of $NO_2$ to each selected meteorology parameters. More meteorology parameters or more complicated functions to describe the dependence of atmospheric $NO_2$ on meteorology conditions deserve further studies as well.

## Data and Methods

### Data

The standard product of tropospheric $NO_2$ column density retrieved from Ozone Monitoring Instrument (OMI) onboard Aura satellite (OMNO2, Level 2, version 003, available at https://disc.gsfc.nasa.gov/datasets/OMNO2_003/summary) was used in this study. Original orbit data were gridded into 0.25*0.25 degree to collocate with reanalysis data. To exclude the potential impacts from cloud contamination, only samples with cloud fraction <30% and $NO_2$ column concentration $<50\times10^{15}$ molec/cm$^2$ were used in our study. Row anomaly issue was carefully treated by using the official quality flag of OMNO2 (see OMNO2 README file) and was based on the abnormal proportion of negative value in the data (if the ratio of negative value in an x-track was larger than 2%, all the data in the track were not used).

The ECMWF atmospheric reanalysis data (ERA5, Single Level and Pressure Level) were used to provide meteorological parameters in this study. Original ERA5 data have a spatial resolution of 0.25*0.25 degree and a temporal resolution of one hour.

Three migration indices derived from Baidu location-based services (LBS) were used to quantify the human mobility. They are proxies for the population moving flow in (immigration index, I-index), out of (emigration index, E-index) the city and inside the city (intra-city index, C-index). The data are operated by Baidu, Inc. and are available at https://qianxi.baidu.com/. The information is derived from billions of location requested per day using Baidu Map app with the permission of sharing from users. All of the proxies are not absolute number of travelers but proportional values.

ORIGINAL UNEDITED MANUSCRIPT

**Statistical model of atmospheric $NO_2$**

A multiple variable linear regression model was developed to quantify tropospheric nitrogen dioxide ($NO_2$) as a function of meteorological factors, which was inspired by the model described by *de Foy and Schauer* (2015) (27) and *Seo J.* et. al. (2018) (31) , using combined satellite observations and atmosphere reanalysis data. Based on our statistics on the satellite retrievals (refer to **Figure S17**) and the studies (28, 39), the values of atmospheric column $NO_2$ are log-normally distributed. Therefore, we used log($NO_2$) to do the multiple regression analysis so that we can scale to a normal distribution with zero mean and unit standard deviation. Contributions from emission source are assumed unchanging with time in this model. The tropospheric $NO_2$ is considered as a linear function of five key meteorological factors (25), planetary boundary layer height (PBLH), solar radiation (SR), surface temperature (T), relative humidity (RH) and wind speed (WS). To ensure all variables having similar order of magnitude, a logarithm transformation is conducted on $NO_2$ column density, PBLH and SR. As a result, the regression model can be expressed as follows.

$$\log(\mathrm{NO_2}) = \mathrm{b_0} + b_1 \cdot \log(PBLH) + b_2 \cdot \log(SR + 10) + b_3 \cdot T + b_4 \cdot RH + b_5 \cdot WS$$

The coefficients of $b_0$ to $b_5$ are regression coefficients determined by Iterative Reweighted Least Squares (IRLS) fitting method (27). We used data of 2018 and 2019 from 45 days before to 65 days after the Chinese Spring Festival (Chinese New Year based on Lunar Calendar) as training data to build up the regression model and used 2017 data to test the model and assessed the modelling error. Then we extended the model with estimated error to predict the column $NO_2$ density in year 2020 assuming there were no changes of anthropogenic emission. For more discussions about the development of the model please refer to the **Supplementary Materials**.

**GEOS-Chem model simulation**

The GEOS-Chem chemical transport model (http://www.geos-chem.org, version 12-8-1) is driven by assimilated meteorological data of MERRA-2 with nested 0.5°×0.625° horizontal resolution. The GEOS-Chem model includes fully coupled $O_3$-NOx-VOC-halogen-aerosol chemistry. The chemical boundary conditions are updated every three hours from a global simulation with 4°×5° resolution. The model has been used to investigate $O_3$ changes in China in recent literatures (40, 41). Emissions in GEOS-Chem are computed by the Harvard-NASA Emission Component (HEMCO). Global default anthropogenic emissions are from CEDS (Community Emissions Data system) (42). Regional emissions are replaced by MEIC (Multiresolution Emission Inventory for China) in China, MIX in other regions of Asia (38). The total anthropogenic NOx and VOCs emission in MEIC inventory are further scaled based on public literatures (12, 43) to obtain the annual emission in 2019. Open fire emissions are from the Global Fire Emission Database (GFED4) (44). Natural emissions of $O_3$ precursors, including NOx from lightning and soil and VOCs from vegetation are calculated on the basis of the assimilated MERRA-2 meteorology. The biogenic emissions of VOCs are calculated according to the Model of Emission of Gases and Aerosols from Nature (MEGAN v2.10) (45).

ORIGINAL UNEDITED MANUSCRIPT

## Data availability

$NO_2$ data are available at https://disc.gsfc.nasa.gov/datasets/OMNO2_003/summary. The ERA5 meteorological data are from https://www.ecmwf.int/en/forecasts/datasets/reanalysis-datasets/era5. The migration data are from https://qianxi.baidu.com/.

## Code availability

The computer codes used to analyze the data are available from the corresponding author on reasonable request.

## Acknowledgement

We deeply appreciate the constructive comments from three anonymous reviewers, which helped us to improve the scientific quality of this study. And we are deeply grateful to Zhifeng Liu from USTCIF (University of Science and Technology of China Initiative Foundation) and Hengshu Zhu from Baidu Inc. for their help. The numerical calculations in this paper have been done on the supercomputing system in the Supercomputing Center of University of Science and Technology of China.

## Funding

This work was supported by the National Key Research and Development Program of China (2017YFC1501402), the National Natural Science Foundation of China (41830104, 41661144007 and 41675022), the "Hundred Talents Program" of the Chinese Academy of Sciences, and the Jiangsu Provincial 2011 Program (Collaborative Innovation Center of Climate Change).

## Author contributions

R. Li. conceived the idea and guided the whole study. R. Li, Y. Zhang and H. Bo designed the experiment. Y. Zhang, H. Bo, Z. Jiang, J. Chen and Y. Wang conducted the research. All authors contributed through discussions and interpretations of the results. R. Li wrote the paper with subsequent inputs from all the co-authors.

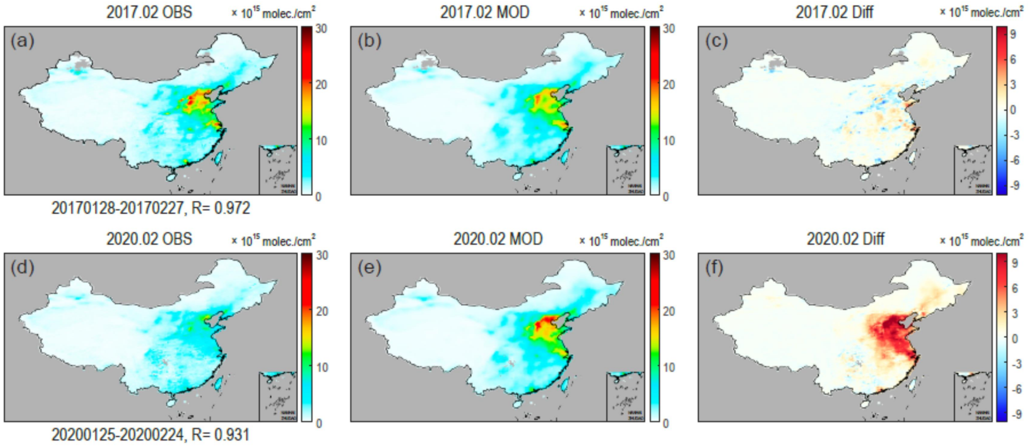

**Figure 1**. The monthly mean (one month after Chinese Spring Festival) of observed $NO_2$ (OBS), modelled $NO_2$ (MOD), and their difference (MOD - OBS) in atmosphere in 2017 and 2020. R is the spatial correlation between OBS and MOD.

ORIGINAL UNEDITED MANUSCRIPT

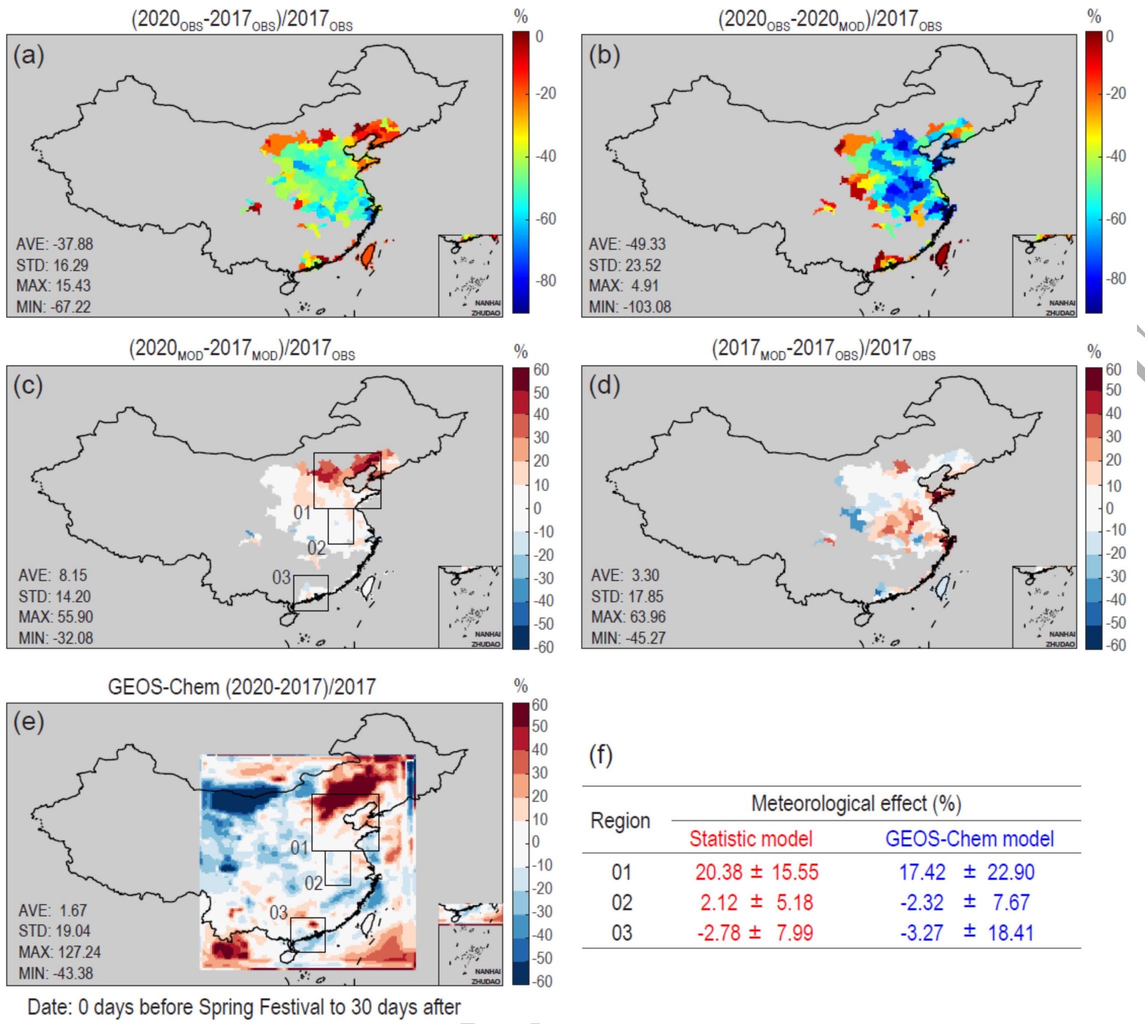

| Region | Meteorological effect (%) Statistic model | Meteorological effect (%) GEOS-Chem model |
|---|---|---|
| 01 | 20.38 ± 15.55 | 17.42 ± 22.90 |
| 02 | 2.12 ± 5.18 | -2.32 ± 7.67 |
| 03 | -2.78 ± 7.99 | -3.27 ± 18.41 |

**Figure 2.** The isolated contribution of emission and meteorology to the changes of $NO_2$ between 2020 and 2017 in 163 China's cities with monthly mean $NO_2$ over $3\times10^{15}$molec/cm$^2$. (a) Relative reduction of satellite observed atmospheric $NO_2$ (%) (b) Estimated emission induced reduction of $NO_2$ (%); (c) Estimated weather induced changes of $NO_2$ (%); (d) Modelling error of the estimation (%); (e) GEOS-Chem model simulated weather induced changes of $NO_2$ (%); (f) The mean and spatial variations of weather induced changes of $NO_2$ in the three selected regions (marked in c and e) from statistic model and GEOS-Chem are listed in the table in the lower-right.

ORIGINAL UNEDI

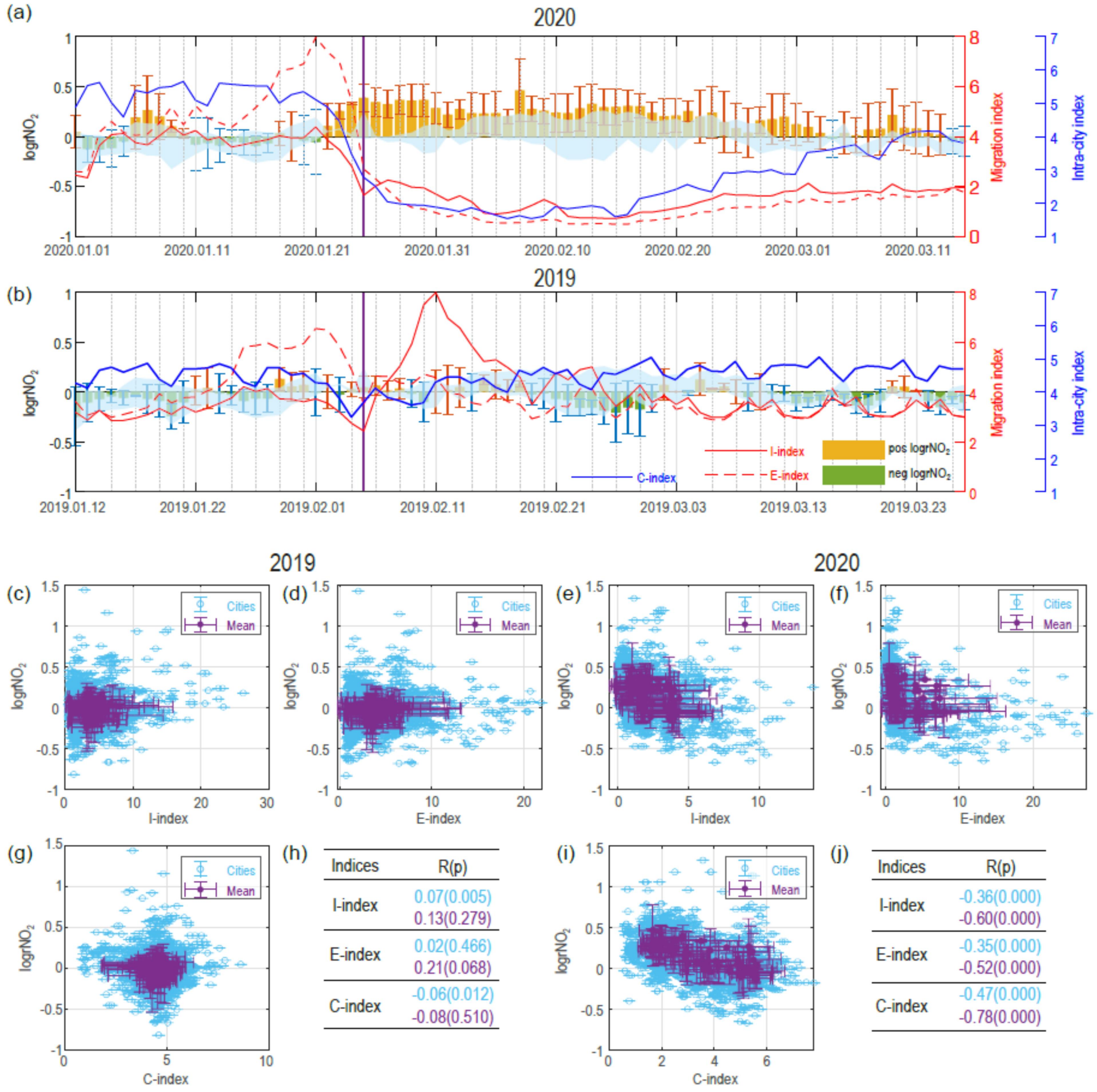

**Figure 3**. Upper panel: The time series of daily logr$NO_2$ (vertical bars), I-index (red curves), E-index (dash red curves), C-index (blue curves) averaged in megacities with population over 8 million in (a) 2020 and (b) 2019 around the Spring Festival (the purple vertical lines).The light blue shadows represent multiple year (2017-2019) mean $NO_2$ with one standard deviation ($\pm\sigma$). Lower panel: Scatter plots of daily logr$NO_2$ to (c) I-index in 2019; (d) E-index in 2019; (e) I-index in 2020; (f) E-index in 2020; (g) C-index in 2019; (h) Correlation Coefficients R and p-values (in parenthesis) in 2019; (i) C-index in 2020; (j) Correlation Coefficients R and p-values (in parenthesis) in 2020. Each blue circle presents the information in one city on each day. Each purple circle presents averaged information of all the selected cities on each day.

ORIGINAL UN

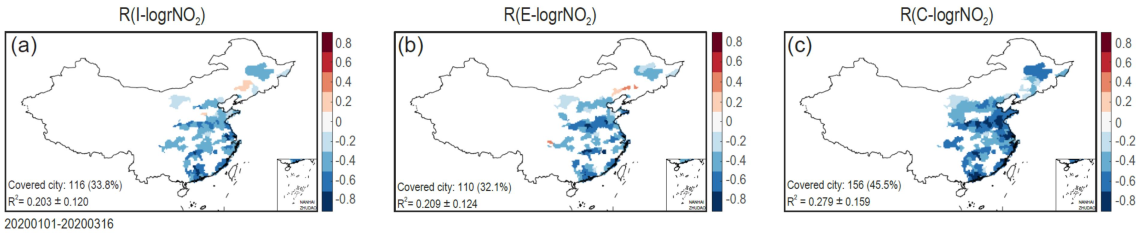

**Figure 4**. The spatial distribution of the correlation coefficients between logr$NO_2$ and the human mobility indices of (a) I-index; (b)E-index and (c) C-index in Chinese mainland. Only cities with mean $NO_2$ over $3\times10^{15}$ molec/cm$^2$ during 2019 Jan 12$^{th}$ to Mar 27th and passed the 95% significance test are filled with colors. The number included in the term 'Covered city' represents the number and the associated percentage of cities showing negative correlations between logr$NO_2$ and the human mobility indices.

ORIGINAL UNEDITED MANUSCRIPT

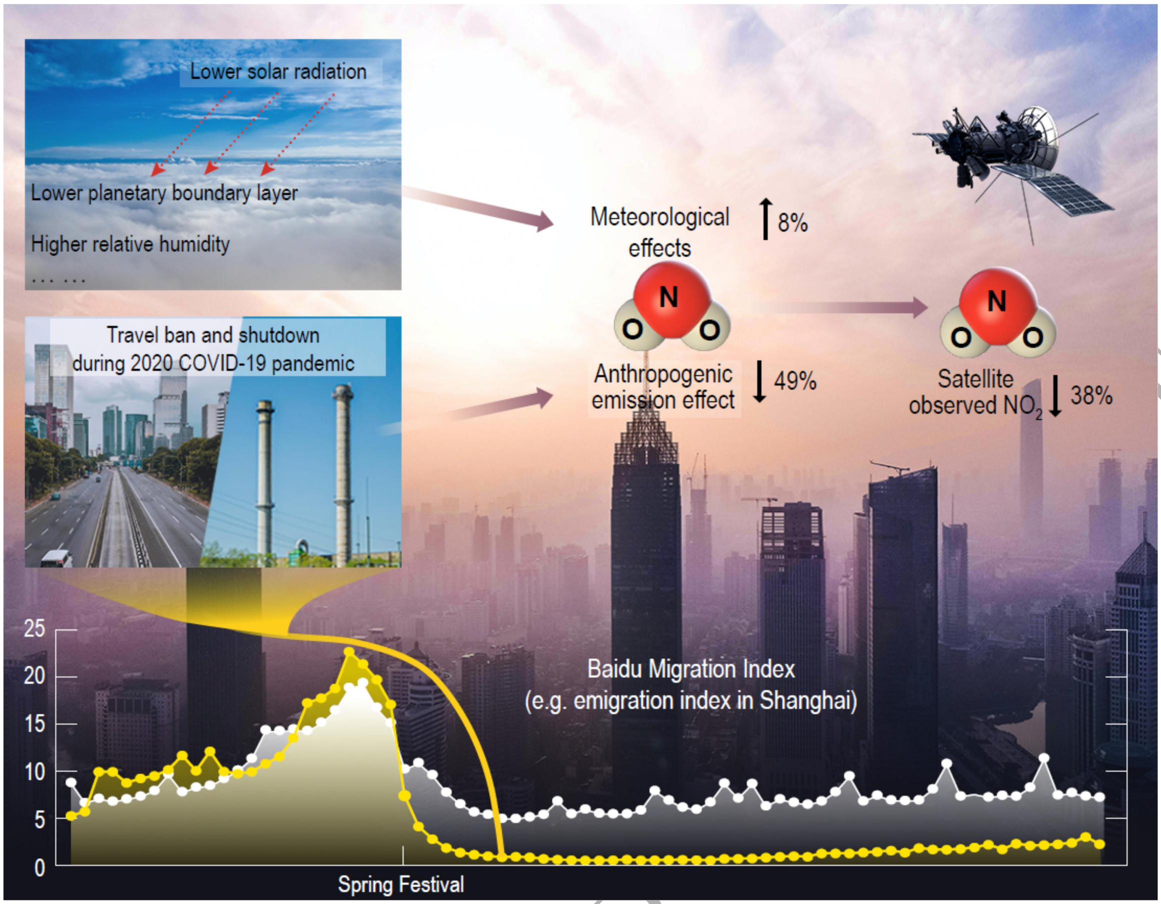

**Figure 5**. Conceptual plot showing the combined meteorological effect and anthropogenic emission effect on the satellite observed $NO_2$ in China during the COVID-19 pandemic in early 2020. The curves at the bottom show the time series of daily Badu migration index (move-out index) in Shanghai during the period around Chinese Spring Festival in 2019 (white curve) and 2020 (yellow curve). The data are operated by Baidu Inc.

ORIGINAL UNEDITED MANUSCRIPT

**Table 1.** The explained variance of emission based reduction of $NO_2$ by the migration indices of C-index $R^2$(C), the I-index $R^2$(I), and the E-index $R^2$(E) in 29 megacities each with population of over 8 million. The cities are sorted by the $R^2$(C) from large to small.

| City | $R^2$(C) | $R^2$(I) | $R^2$(E) |
|---|---|---|---|
| Dongguan | 0.708 | 0.145 | 0.312 |
| Guangzhou | 0.667 | 0.626 | 0.366 |
| Xuzhou | 0.665 | 0.325 | 0.410 |
| Suzhou | 0.658 | 0.412 | 0.218 |
| Heze | 0.641 | 0.207 | 0.317 |
| Shangqiu | 0.594 | 0.277 | 0.333 |
| Shenzhen | 0.563 | 0.133 | 0.292 |
| Linyi | 0.532 | 0.195 | 0.248 |
| Xi'an | 0.451 | 0.298 | 0.117 |
| Jinhua | 0.449 | 0.289 | 0.232 |
| Ganzhou | 0.437 | 0.477 | NaN |
| Jining | 0.433 | 0.149 | 0.325 |
| Nanjing | 0.414 | 0.158 | NaN |
| Quanzhou | 0.395 | 0.344 | 0.319 |
| Weifang | 0.381 | 0.113 | 0.089 |
| Tianjin | 0.331 | 0.198 | 0.093 |
| Zhoukou | 0.315 | 0.114 | 0.273 |
| Zhumadian | 0.304 | 0.130 | 0.292 |
| Baoding | 0.292 | 0.141 | 0.176 |
| Harbin | 0.288 | 0.198 | 0.178 |
| Shijiazhuang | 0.266 | 0.133 | 0.118 |
| Chongqing | 0.255 | 0.232 | 0.227 |
| Beijing | 0.248 | NaN | 0.080 |
| Wuhan | 0.248 | 0.235 | 0.230 |
| Shanghai | 0.142 | NaN | 0.120 |
| Nanyang | 0.141 | NaN | 0.151 |
| Xinyang | 0.087 | 0.077 | 0.090 |
| Chengdu | NaN | NaN | NaN |
| Handan | NaN | 0.081 | NaN |
| AVERAGE | 0.404±0.177 | 0.227±0.132 | 0.224±0.098 |

ORIGINAL UNEDITED MANUSCRIPT
