# Untangling the contributions of meteorological conditions and human mobility to tropospheric NO_2_ in Chinese mainland during the COVID-19 pandemic in early 2020

**DOI:** 10.1093/nsr/nwab061

**Published:** 2021-04-09

**Authors:** Yuxiang Zhang, Haixu Bo, Zhe Jiang, Yu Wang, Yunfei Fu, Bingwei Cao, Xuewen Wang, Jiaqi Chen, Rui Li

**Affiliations:** School of Earth and Space Sciences, University of Science and Technology of China, Hefei 230026, China; Comparative Planetary Excellence Innovation Center, Frontiers Science Center for Planetary Exploration and Emerging Technologies, Chinese Academy of Sciences, Hefei 230026, China; School of Earth and Space Sciences, University of Science and Technology of China, Hefei 230026, China; State Key Laboratory of Fire Science, University of Science and Technology of China, Hefei 230026, China; School of Earth and Space Sciences, University of Science and Technology of China, Hefei 230026, China; School of Earth and Space Sciences, University of Science and Technology of China, Hefei 230026, China; School of Earth and Space Sciences, University of Science and Technology of China, Hefei 230026, China; Jiangxi Ecological Environment Monitoring Center, Nanchang 330000, China; Green Earth Science and Education Service, Slingerlands, NY 12259, USA; School of Earth and Space Sciences, University of Science and Technology of China, Hefei 230026, China; School of Earth and Space Sciences, University of Science and Technology of China, Hefei 230026, China; Comparative Planetary Excellence Innovation Center, Frontiers Science Center for Planetary Exploration and Emerging Technologies, Chinese Academy of Sciences, Hefei 230026, China; State Key Laboratory of Fire Science, University of Science and Technology of China, Hefei 230026, China

**Keywords:** atmospheric nitrogen dioxide, anthropogenic emissions, meteorology conditions, human mobility, COVID-19 quarantine

## Abstract

In early 2020, unprecedented lockdowns and travel bans were implemented in Chinese mainland to fight COVID-19, which led to a large reduction in anthropogenic emissions. This provided a unique opportunity to isolate the effects from emission and meteorology on tropospheric nitrogen dioxide (NO_2_). Comparing the atmospheric NO_2_ in 2020 with that in 2017, we found the changes of emission have led to a 49.3 ± 23.5% reduction, which was ∼12% more than satellite-observed reduction of 37.8 ± 16.3%. The discrepancy was mainly a result of changes of meteorology, which have contributed to an 8.1 ± 14.2% increase of NO_2_. We also revealed that the emission-induced reduction of NO_2_ has significantly negative correlations to human mobility, particularly that inside the city. The intra-city migration index derived from Baidu Location-Based-Service can explain 40.4% ± 17.7% variance of the emission-induced reduction of NO_2_ in 29 megacities, each of which has a population of over 8 million in Chinese mainland.

## Teaser

The tangled effects from anthropogenic emission and meteorology condition on atmospheric NO2 in China was quantitatively isolated using satellite remote sensing, numerical modeling and traveling big data during the unprecedented COVID-19 related travel bans and lockdowns in early 2020.

## INTRODUCTION

To curb the spread of COVID-19, the Chinese government implemented nationwide strict control measures from late January to March 2020 [1,2]. Lockdowns were imposed in cities and provinces, leading to a gradual cessation in inter-city and inter-province traffic [1]. Inside cities and villages, strict self-quarantine was also implemented. People had to stay at home, except for shopping for necessities or seeking medical treatment. Businesses and industries suspended operations or largely reduced production. The intensive lockdown measures led to a dramatic decrease in human mobility [3,4].

Nitrogen dioxide (NO_2_), as one of the most important air pollutants, is harmful to the human respiratory system [5–7] and plays essential roles in the formation of acid rains, second order aerosols [8] and ozone [9–11]. The dominant sources of tropospheric NO_2_ over east China are anthropogenic combustions in winter, of which the contributions from power generation, industry and transportations are about 19%, 42% and 35%, respectively [12]. The decrease in human mobility resulting from the lockdown measures is expected to have produced impacts on tropospheric NO_2_ via effects on industry and transportation activities [13,14]. Recent studies have reported a satellite-observed large drop in column NO_2_ density during this period because of the COVID-19 quarantine [1,13,15].

Besides anthropogenic emissions, tropospheric NO_2_ concentrations are also strongly modulated by changes in meteorological conditions [16–19]. Changes in wind speed, atmosphere stability (related to temperature and pressure etc.), solar radiation and humidity from day to day can quickly change the atmospheric NO_2_ densities [16,20]. Temperature and humidity are crucial to the photochemical processes related to NO_2_ [21]. Higher temperature and higher humidity can reduce the lifetime of NO_2_ and accelerate the conversion of NO_2_ to secondary nitrate aerosols [17,19], thus resulting in a negative correlation with atmospheric NO_2_ concentration in most places [16,19]. Solar radiation is the key factor controlling the photodissociation rate of NO_2_ (NO_2_→NO + O), and can greatly affect the lifetime of NO_2_ [21,22]. This is strongly supported by observation of increased NO_2_ concentration during the solar eclipse [22]. In general, surface NO_2_ concentration is found to decrease with increasing solar radiation [16,21]. In addition, high wind speed and high planetary boundary layer height (PBLH) both favor dispersion and dilution of air pollutants in the boundary layer of the atmosphere [16,17] and can reduce NO_2_ concentration [21,23,24].

Anthropogenic emissions and meteorological conditions can both affect atmospheric NO_2_ concentration, but their effects are often tangled. Although the reported literature demonstrates the important influences of lockdowns on tropospheric NO_2_ [13,25], the respective contributions from anthropogenic and meteorological processes are not clear.

Chemical transport models can be used to analyze the sources of atmospheric composition changes. However, the modelled results can be affected by potential uncertainties in the emission and chemistry processes. For example, Liu *et al.* showed that the modelled surface NO_2_ over North China Plain is about 34% lower than surface measurement with the GEOS-Chem model, but is 26% higher using the CMAQ model [26]. Recent studies suggest the possibility of constraining the observation-based anthropogenic and meteorological influences with statistical models to avoid the effects of potential uncertainties in model simulations [27–29]. During the early stages of the COVID-19 pandemic, anthropogenic emissions in China were much lower than before [13,15], while changes in meteorology conditions in 2020 were expected to be smaller than changes in emissions. This provides an ideal test-bed to study the separate impacts of emission and meteorological changes on atmospheric NO_2_ with statistical models.

In this study, we investigated the effects of meteorology conditions and human mobility associated with COVID-19 quarantine on atmospheric NO_2_ in China using a statistical model to represent the NO_2_ [27]. The human mobility strengths, including migration and intra-city flow were quantified using Baidu Migration data [3,30]. We focused on the month before (hereafter Month-01) and the month after (hereafter Month-02) the Chinese Spring Festival in 2017, 2018, 2019 and 2020 to take the holiday effect on human mobility into account.

## RESULTS

### Statistical model of troposphere NO_2_

According to the annual Report on the State of the Environment in China from 2015 to 2019 (http://english.mee.gov.cn/Resources/Reports/soe/), the mean NO_2_ concentrations of the cities in China were relatively stable from 2017 to 2019. Before that, anthropogenic NOx emissions (normalized in 2010) were reduced by about 21% in 2012–2015 (7%/year) and about 6% in 2015–2017 (3%/year) [12]. The dramatic declines of anthropogenic NOx emissions in 2012–2015, as well as the subsequent slowdown of emission reductions, were mainly driven by installation of selective catalytic reduction (SCR) systems in utilities for coal-fired power plants [12].

Recent studies have revealed that the satellite-observed column NO_2_ density in China in 2020 Month-02 was much lower than that in 2019 [15], and concluded that this drop was attributed to the COVID-19 related city lockdowns and travel bans [1]. However, it must be recognized that the atmospheric NO_2_ concentration is also greatly affected by meteorology conditions [15].

Assuming the real measurements of atmospheric NO_2_ (in logarithm) can be separated into two parts, NO_2_ contributed by emission ${{\rm{F}}_{Emis}}( {{\rm{x}},{\rm{t}}} )$ and by meteorology conditions ${{\rm{G}}_{Mete}}( {{\rm{x}},{\rm{t}}} )$, we have the following function associated with geolocation (x) and time (t) [27]:

(1) \begin{eqnarray*}{\rm{log\ N}}{{\rm{O}}_2}{\left( {{\rm{x}},{\rm{t}}} \right)^{OBS}} &=& {{\rm{F}}_{Emis}}{\left( {{\rm{x}},{\rm{t}}} \right)^{OBS}}{\rm{\ }}\nonumber \\ &&+ {\rm{\ }}{{\rm{G}}_{Mete}}{\left( {{\rm{x}},{\rm{t}}} \right)^{OBS}}.\end{eqnarray*}

A statistical model of ${\rm{logN}}{{\rm{O}}_2}{( {{\rm{x}},{\rm{t}}} )^{MOD}}$ was established on two assumptions. (i) The regression model was trained in Month-01 and Month-02 in 2018 and 2019. Considering the fairly stable NOx emission in China in recent years [12], ${{\rm{F}}_{Emis}}( {{\rm{x}},{\rm{t}}} )$ based on 2018 and 2019 should provide a good approximation for the anthropogenic influences for 2017 and 2020. Thus, we assumed the temporal variations of ${{\rm{F}}_{Emis}}( {{\rm{x}},{\rm{t}}} )$ were negligible at each grid of 0.5 $\times $ 0.5 degree. (ii) The temporal and spatial variations of ${{\rm{G}}_{Mete}}( {{\rm{x}},{\rm{t}}} )$ can be modelled using a simplified linear function of five key meteorology parameters as described in Data and methods. We found these two assumptions resulted in good agreement between the observed and modelled tropospheric NO_2_, based on self-consistency check (using training data) and independent check (using independent data). Therefore, we have:

(2) \begin{eqnarray*}{\rm{log\ N}}{{\rm{O}}_2}{\left( {{\rm{x}},{\rm{t}}} \right)^{MOD}} &=& {{\rm{F}}_{Emis}}{\left( {\rm{x}} \right)^{MOD}}{\rm{\ }}\nonumber \\ &&+\ {{\rm{G}}_{Mete}}{\left( {{\rm{x}},{\rm{t}}} \right)^{MOD}}.\end{eqnarray*}

It should be noted that the modelled emission term ${{\rm{F}}_{Emis}}{( {\rm{x}} )^{MOD}}$ in Eq. (2) is only a function of geolocation. In other words, its value stays constant at given 0.5 $\times $ 0.5 degree box based on statistical regression. The detailed regression procedures and sensitivity tests are described in Data and methods. The difference between satellite observations and modelling results can be expressed as:

(3) \begin{eqnarray*} {\rm{Log }}\left[ {\frac{{{\rm{N}}{{\rm{O}}_2}{{\left( {{\rm{x}},{\rm{t}}} \right)}^{MOD}}}}{{{\rm{N}}{{\rm{O}}_2}{{\left( {{\rm{x}},{\rm{t}}} \right)}^{OBS}}}}} \right] &=& {\rm{\ }}\big[ {{\rm{F}}_{Emis}}{{\left( {{\rm{x}},{\rm{t}}} \right)}^{MOD}}\nonumber \\ &&-\ {{\rm{F}}_{Emis}}{{\left( {\rm{x}} \right)}^{OBS}} \big] + {\rm{\Delta \ }},\nonumber \\ \end{eqnarray*}

where the first term at the right hand of Eq. (3) represents the error introduced by ignoring the temporal variations of emission. The second term Δ represents the modelling error of ${{\rm{G}}_{Mete}}( {{\rm{x}},{\rm{t}}} )$.

**Figure 1.**
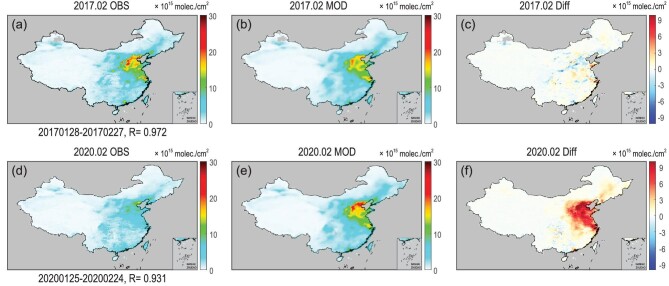
The monthly mean (1 month after the Chinese Spring Festival) of observed NO_2_ (OBS) (a and d), modelled NO_2_ (MOD) (b and e), and their difference (MOD − OBS) (c and f) in the atmosphere in 2017 and 2020. R is the spatial correlation between OBS and MOD.

The performance of the model was analyzed using independent (from establishing the model) observations in 2017. The model successfully predicted the monthly mean atmospheric NO_2_ in 2017 Month-02 (Fig. 1), with negligible bias in most areas in China. Even in the heaviest polluted areas in central and eastern China with NO_2_ over 10 $\times $10^15^ molec/cm^2^, the mean bias is only 4.3% compared with satellite observations. On the other hand, the spatial correlation coefficients between model prediction and satellite NO_2_ are as high as 0.97 (*P* < 0.001). Similar results for 2018 and 2019 can be seen in Fig. S4.

The model also works well at predicting daily NO_2_. At the scales of provinces (Fig. S5) and cities (Fig. S6), statistically significant positive correlation coefficients between daily mean NO_2_ predicted by the model and the real satellite measurements can be found in most areas in the mainland of China in Month-01 and Month-02 in 2017, 2018 and 2019.

The above results demonstrate that the contribution of emission to the atmospheric NO_2_, in the same month of the adjacent years, can be estimated as a time-independent geolocation-based function. The spatial and temporal variations of meteorology effects can be modelled using the linear function of five selected key parameters. The modelling error is generally <5% based on validations in 2017. The reason for the emission situation in 2017 was similar to that in 2018/2019, and the regression model captured the quantitative dependence of atmospheric NO_2_ on meteorology conditions. Consequently, if the anthropogenic emissions in 2020 were similar to those in 2018/2019 (i.e. without the effects from COVID-19 quarantine), the model was expected to provide good prediction for tropospheric NO_2_ in 2020.

It should be noted that changes in column atmospheric NO_2_ are not linearly associated with emissions because of nonlinear effects from atmospheric chemistry. Based on results from the GEOS-Chem chemical transport model study (Fig. S17), we found a broadly linear response of modelled tropospheric NO_2_ columns to changes in anthropogenic NOx and VOCs emissions, that is 50% reduction of anthropogenic emissions results in about 45% reduction of tropospheric NO_2_ columns. The model simulations suggest that the influence from nonlinear processes is small (about 5%).

### Quarantine-induced reduction in troposphere NO_2_

In 2020, although NO_2_ variations related to meteorology conditions could still be modelled with good accuracy, the emissions of NO_2_ were significantly reduced because of the city lockdowns and travel bans. Therefore, the foundation of the statistical model describing the contribution from emissions collapsed. The term ${{\rm{F}}_{Emis}}{( {{\rm{x}},{\rm{t}}} )^{OBS}} - {{\rm{F}}_{Emis}}{( {\rm{x}} )^{MOD}}$ in Eq. (3) in 2020 became much larger than that in 2017. The model overestimated monthly mean NO_2_ by 6–9 $\times $ 10^15^molec/cm^2^ in the heavily polluted areas in China (Fig. 1d). Similar overestimation also could be seen from the time series of daily mean NO_2_ in most polluted cities and provinces (Figs S7–S10), such as Tianjin, Shanghai, Shandong, Jiangsu and Beijing.

**Figure 2.**
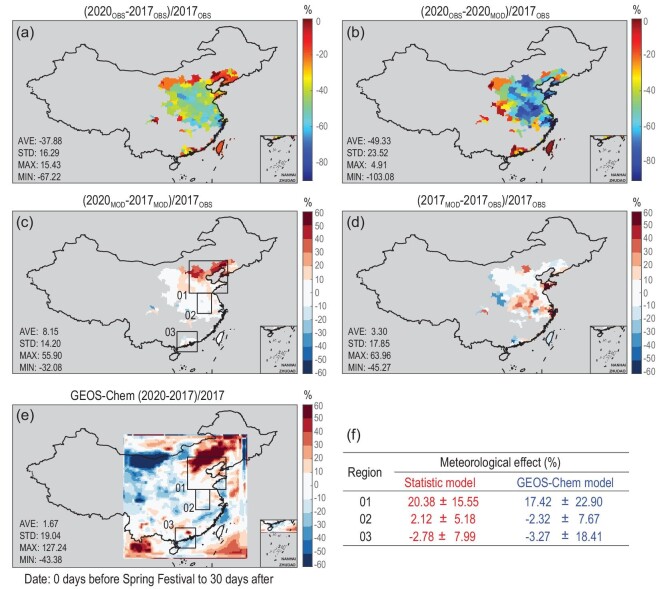
The isolated contribution of emission and meteorology to the changes of NO_2_ between 2020 and 2017 in 163 cities in China with monthly mean NO_2_ over 3 $\times $ 10^15^ molec/cm^2^. (a) Relative reduction of satellite-observed atmospheric NO_2_ (%). (b) Estimated emission-induced reduction of NO_2_ (%). (c) Estimated weather-induced changes of NO_2_ (%). (d) Modelling error of the estimation (%). (e) GEOS-Chem model simulated weather-induced changes of NO_2_ (%). (f) The mean and spatial variations of weather-induced changes of NO_2_ in the three selected regions (marked in c and e) from the statistical model and GEOS-Chem are listed in the table.

If we compare the satellite observation of atmospheric NO_2_ in 2020 with that in 2017, we could decompose the difference into three isolated terms [31]:

(4) \begin{eqnarray*} &&\!\!\!\!{\rm{N}}{{\rm{O}}_2}{\left( {{\rm{x}},2020} \right)^{OBS}} - {\rm{N}}{{\rm{O}}_2}{\left( {{\rm{x}},2017} \right)^{OBS}}\nonumber\\ && \!= \big[ {{\rm{N}}{{\rm{O}}_2}{{\left( {{\rm{x}},2020} \right)}^{OBS}} - {\rm{N}}{{\rm{O}}_2}{{\left( {{\rm{x}},2020} \right)}^{MOD}}} \big]\nonumber\\ &&\quad\!\!\!+\, \big[ {{\rm{N}}{{\rm{O}}_2}{{\left( {{\rm{x}},2020} \right)}^{MOD}} - {\rm{N}}{{\rm{O}}_2}{{\left( {{\rm{x}},2017} \right)}^{MOD}}} \big]\nonumber\\ &&\quad\!\!\!+\, \big[ {{\rm{N}}{{\rm{O}}_2}{{\left( {{\rm{x}},2017} \right)}^{MOD}} - {\rm{N}}{{\rm{O}}_2}{{\left( {{\rm{x}},2017} \right)}^{OBS}}} \big],\nonumber\\ \end{eqnarray*}

where the first term represents the emission-induced reductions in 2020; the second represents the meteorology induced variations; and the third represents the modelling error. Using real satellite observations and modelling results in 2020 and 2017, the map of the contributions (unit: %) from emission and meteorology (i.e. the above three terms) to the reduction of NO_2_ is shown in Fig. 2. In most of China's cities with monthly mean NO_2_ over 3 $\times $ 10^15^ molec/cm^2^, we found that emission-induced reductions in 2020 (Fig. 2b) were larger than the satellite-observed reductions (Fig. 2a), because the meteorology in 2020 led to a net increase of NO_2_ compared to 2017 (Fig. 2c).

Overall, the Ozone Monitoring Instrument (OMI) observed troposphere NO_2_ decreased by 37.8 ± 16.3% in 2020 from that in 2017. Using the model mentioned above, we estimated that if the weather conditions in 2020 were the same as that in 2017, the NO_2_ columns should have decreased by 49.3 ± 23.5% because of the reduced anthropogenic emission, which means the emission-induced reduction of NO_2_ resulting from the COVID-19 quarantine was actually higher than OMI actual observations. The meteorological conditions in 2020 did not favor the dilution and ventilation of air pollutants, and thus led to an increase of NO_2_ of 8.1 ± 14.2%. Meanwhile, the modelling error of the above estimation is only 3.32 ± 17.8%, which is significantly smaller than the other two terms.

The statistic model results are consistent with GEOS-Chem model simulations. As shown in Fig. 2e, there are good agreements in the derived impacts of meteorological variability. Both GEOS-Chem-based and statistics-based results show positive contributions from meteorological variability in northern China, and neutral and weakly negative contributions in central and southern China. Quantitatively, the weather effects from GEOS-Chem- and statistics-based analysis are 17.42 ± 22.90% versus 20.38 ± 15.55% over the selected North China Plain area, −2.32 ± 7.67% versus 2.12 ± 5.18% in Anhui and −3.27 ± 18.41% versus −2.78 ± 7.99% in Guangdong.

The results are also consistent with other studies in the literature [14,32–34]. Zhang *et al.* estimated the daily NOx emission in 2020 by combining TROPOMI NO_2_ observation with WRF-GEOSChem simulations, and reported a 50% decrease of emission after the COVID-19 lockdown [14]. Marlier *et al.* observed a 49% decline of NO_2_ after the Lunar New Year, and found the weather conditions weakened the emission reduction [33]. Zhao *et al.* applied the emission inventory of 2017 to WRF-CMAQ, and concluded that the meteorological condition in 2020 elevated the NO_2_ concentration in over half of the cities in China [34]. Wang *et al.* found the reduction of PM2.5 simulated by WRF-CMAQ is smaller than the reduction of precursor emissions, also indicating the unfavorable meteorology (lower PBLH, WS and higher RH) for the dilution of the pollutants [35].

Therefore, the COVID-19 quarantine actually has caused a reduction of NO_2_ larger than that seen from the satellite observation (i.e. a direct comparison of 2020 with 2017), but the weather effect has cancelled out some of the emission effect. The modelling error is significantly smaller than each of the two effects, particularly the mean value. This is the first time contributions of emission and weather to the satellite-observed reduction of NO_2_ in early 2020 have been isolated. Similar analyses using situations in 2018 and 2019 are given in the Supplementary data (Figs S11 and S12). Overall, when comparing 2020 with 2018 and 2019, the emission-induced reduction of NO_2_ was also significantly larger than the satellite-observed reduction from the meteorology contribution. This confirmed the conclusion derived from the 2020–2017 comparison.

### Human mobility and NO_2_

Based on Eq. (3), the difference between satellite observations and model prediction in 2020, i.e. ${\rm{logrN}}{{\rm{O}}_2} ( {2020} ) = {\rm{logN}}{{\rm{O}}_2}{( {\rm x,2020} )^{MOD}} - {\rm{logN}}{{\rm{O}}_2}{( {\rm x,2020} )^{OBS}},$ mainly represents the emission-related reduction of NO_2_ (hereafter logrNO_2_), which can be attributed to multiple factors including the prohibition of human mobility implemented by the government, closure of businesses consuming fossil fuels, such as restaurants, hotels etc., reduction of industrial production because of weakened domestic and international trades etc. It is hard to make a thorough survey to measure all of those factors in the current situation when COVID-19 remains a serious threat to human health.

Fortunately, satellite location-based services (LBS) describing human mobility [36] are useful proxies of anthropogenic emissions. To what extent can the emission-related reduction of NO_2_ in 2020 be explained using LBS data? To quantitatively understand the roles of city lockdowns and travel bans played in reducing the air pollutants in China, we investigated the correlations between the emission-related reduction of NO_2_ and Baidu migration data (Data and methods) of three indices representing the relative population flow moving in (I-index), moving out (E-index) and moving inside the city (C-index). The spatial patterns of the indices in the mainland of China in Month-01 and Month-02 in 2019 and 2020 are shown in Fig. S13. Generally, values are higher in eastern China than those in western China. The spatial patterns and inhomogeneities are associated with population density and economy activities. For example, megacities such as Beijing, Shanghai, and Guangzhou showed large values of all indices because of their dense population, large numbers of migrant workers and prosperous economy.

**Figure 3.**
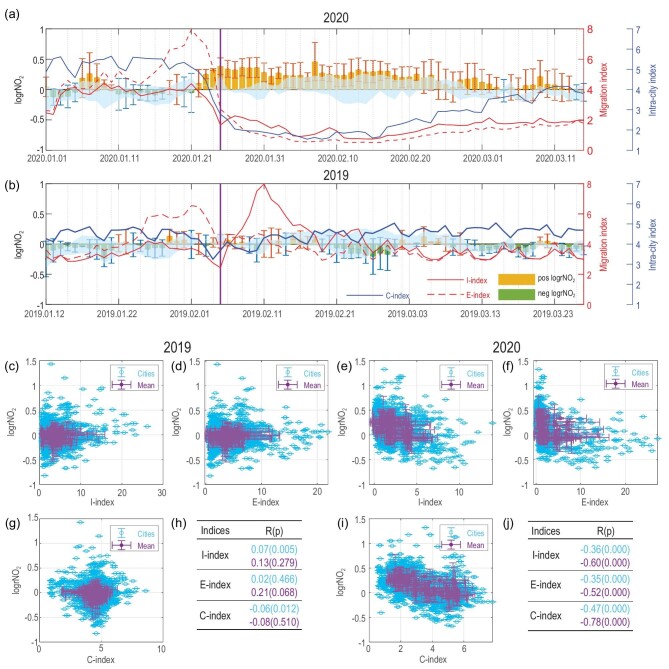
Upper panel: The time series of daily logrNO_2_ (vertical bars), I-index (red curves), E-index (dash red curves), C-index (blue curves) averaged in megacities with a population over 8 million in (a) 2020 and (b) 2019 around the Spring Festival (the purple vertical lines). The light blue shadows represent multiple year (2017–2019) mean NO_2_ with 1 standard deviation (±σ). Lower panel: Scatter plots of daily logrNO_2_ to (c) I-index in 2019; (d) E-index in 2019; (e) I-index in 2020; (f) E-index in 2020; (g) C-index in 2019; (h) correlation coefficients R and *P* values (in parenthesis) in 2019; (i) C-index in 2020; (j) correlation coefficients R and *P* values (in parenthesis) in 2020. Each blue circle presents the information in one city on each day. Each purple circle presents averaged information of all the selected cities on each day.

As shown in Fig. 3, as people started to go home for family reunions in Month-01 2020, the immigration index (I-index, red curves) and emigration index (E-index, dash red line) in megacities with a population over 8 million rapidly increased and peaked around 23 January 2020 when Wuhan, Hubei, was locked down because of a COVID-19 outbreak. After that, both I-index and E-index decreased sharply and remained at very low values during Month-02 2020 [4]. In Month-01, shopping and visiting inside cities also increased in preparation for the coming Spring Festival, and the intra-city C-index (blue curves) was also high before 23 January 2020. For the same reason, the C-index dropped after the lockdowns. The ‘Spring Festival Effect’ was also shown in 2019, except that the C-index picked up quickly several days after the Spring Festival.

Meanwhile, the emission-related change of logrNO_2_ (vertical bars in Fig. 3) in 2020 just oscillated around zero each day before 23 January. After that, it stayed at positive values and was significantly larger than the standard variations. In contrast, in 2019, the difference between satellite observation and model prediction of NO_2_ (ie, logrNO_2_), remained at small values during the whole period of Month-01 and Month-02, and the temporal variations were always within the range of standard deviation. The difference between 2020 and 2019 confirmed that the emission changes in 2020 induced a large reduction in NO_2_.

To investigate the quantitative relationship between logrNO_2_ and the human mobility indices, we constructed scatter plots of daily logrNO_2_ to daily indices in 2019 and 2020 in cities populated over 8 million (lower panel in Fig. 3). In 2019, the correlations between logrNO2 and all migration indices are weak because the emission-induced variations in NO_2_ were very small. While in 2020, daily logrNO_2_ in individual cities or that averaged in all cities both negatively correlated with those migration indices, with *P* < 0.001. The intra-city migration C-index showed the strongest correlation and could explain 22.1% (i.e. the R^2^ at individual city level) to 60.8% (the R^2^ averaged in all megacities) variance of the logrNO_2_. The immigration I-index could explain 13.0% to 36.0%; the emigration E-index could explain 12.3% to 27.0%.

A list of the explained variance of logrNO_2_ by migration indices (from high to low) of all 29 megacities is given in Table 1. In some of the cities in southern China such as Guangzhou and Dongguan, the C-index could explain as much as ∼70% variance. In mid- and eastern China, cities like Suzhou, Heze and Xuzhou also showed explained variance of over 60% by the C-index. This indicates that human mobility inside the city is more important in terms of effects on NO_2_ emissions than the population flow toward (I-index) or out (E-index) of the city.

**Table 1.** The explained variance of emission-based reduction of NO_2_ by the migration indices of C-index R^2^(C), the I-index R^2^(I) and the E-index R^2^(E) in 29 megacities with a population of over 8 million. The cities are sorted by the R^2^(C) from large to small. NaN: insignificant correlation coefficient.

City	R^2^(C)	R^2^(I)	R^2^(E)
Dongguan	0.708	0.145	0.312
Guangzhou	0.667	0.626	0.366
Xuzhou	0.665	0.325	0.410
Suzhou	0.658	0.412	0.218
Heze	0.641	0.207	0.317
Shangqiu	0.594	0.277	0.333
Shenzhen	0.563	0.133	0.292
Linyi	0.532	0.195	0.248
Xi’an	0.451	0.298	0.117
Jinhua	0.449	0.289	0.232
Ganzhou	0.437	0.477	NaN
Jining	0.433	0.149	0.325
Nanjing	0.414	0.158	NaN
Quanzhou	0.395	0.344	0.319
Weifang	0.381	0.113	0.089
Tianjin	0.331	0.198	0.093
Zhoukou	0.315	0.114	0.273
Zhumadian	0.304	0.130	0.292
Baoding	0.292	0.141	0.176
Harbin	0.288	0.198	0.178
Shijiazhuang	0.266	0.133	0.118
Chongqing	0.255	0.232	0.227
Beijing	0.248	NaN	0.080
Wuhan	0.248	0.235	0.230
Shanghai	0.142	NaN	0.120
Nanyang	0.141	NaN	0.151
Xinyang	0.087	0.077	0.090
Chengdu	NaN	NaN	NaN
Handan	NaN	0.081	NaN
AVERAGE	0.404 ± 0.177	0.227 ± 0.132	0.224 ± 0.098

**Figure 4.**
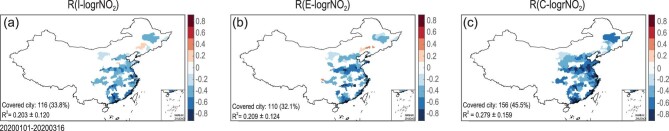
The spatial distribution of the correlation coefficients between logrNO_2_ and the human mobility indices of (a) I-index; (b) E-index and (c) C-index in Chinese mainland. Only cities with mean NO_2_ >3 $\times $ 10^15^ molec/cm^2^ during 12 January to 27 March 2019 that passed the 95% significance test are filled with colors. The number included in the term ‘Covered city’ represents the number and the associated percentage of cities showing negative correlations between logrNO_2_ and the human mobility indices.

Not only in those megacities, but negative correlations between logrNO_2_ and migration indices were also seen nationwide in the mainland of China in 2020. In Fig. 4, among cities with mean NO_2_ over 3 $\times $ 10^15^ molec/cm^2^, the logrNO_2_ showed significantly negative correlations with I-index in 116 cities and with E-index in 110 cities, with explained variance of 0.203 ± 0.120 and 0.208 ± 0.124, respectively. For the C-index, 156 cities showed significantly negative correlations with explained variance of 0.279 ± 0.159. No city showed positive correlation with logrNO_2_ for the C-index. The results indicate that these LBS migration indices, particularly the intra-city index (C-index), to a large extent, provided reasonable explanation for the temporal variations of emission-induced reduction of NO_2_ in large areas of China in early 2020.

## DISCUSSION AND CONCLUSION

The unprecedented lockdowns and travel bans during the COVID-19 lockdown have led to a large reduction in anthropogenic emissions of air pollution [13]. We took the opportunity of this unintentionally conducted circumstance to investigate the isolated effects of emission and meteorology condition on atmospheric NO_2_, and the quantitative relationship between the reductions of NO_2_ and human mobility using state-of-the-art satellite remote sensing products and location-service-based big data. We established a statistical model representing the column density of NO_2_ as a function of only five key meteorology parameters, with the assumption that emission was constant. Compared with satellite observations in early 2017, the model-predicted monthly mean NO_2_ was only biased by 4.3% in the heaviest polluted areas in central and eastern China and showed spatial correlation coefficient of 0.97 (*P* < 0.001).

**Figure 5.**
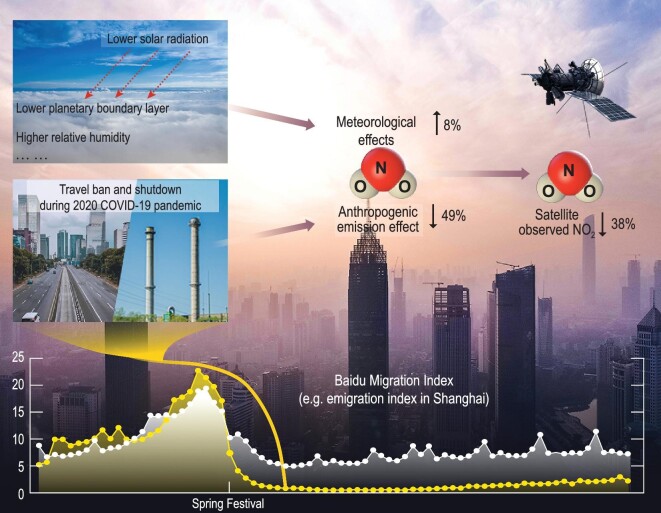
Conceptual plot showing the combined meteorological effect and anthropogenic emission effect on the satellite-observed NO_2_ in China during the COVID-19 pandemic in early 2020. The curves at the bottom show the time series of daily Baidu emigration index in Shanghai during the period around the Chinese Spring Festival in 2019 (white curve) and 2020 (yellow curve). The data are maintained by Baidu Inc.

Using the statistical model, it was found that the travel bans and lockdowns of China in 2020 have resulted in a decrease of observed NO_2_. Meanwhile, changes in meteorological conditions, such as lower PBLH, lower solar radiation etc., have led to an increase of atmospheric NO_2_ (Fig. 5). As shown in Fig. 5, compared with 2017, the anthropogenic emission changes in early 2020 led to a 49.3 ± 23.5% reduction of atmospheric NO_2_, and the changes in meteorological conditions led to an 8.1 ± 14.2% increase. Consequently, the net reduction of NO_2_ was brought down to 37.8 ± 16.3%. The modelling error was 3.3 ± 17.8%. We revealed, for the first time, that the COVID-19 quarantine caused a reduction of atmospheric NO_2_ which was actually larger than what we saw from the satellite observations. In addition, the emission-induced reduction of NO_2_ shows statistically significant correlations to human mobility. Quantitatively, the migration index representing the movement inside the city has the highest explained variance among all indices: it can explain 40.4% ± 17.7% variance on average in 29 megacities with a population of over 8 million in the mainland of China. This study established a method to untangle the contributions of emissions and meteorology conditions to the reduction of atmospheric NO_2_, and quantitatively assessed the effect of the city lockdowns and travel bans on the tropospheric NO_2_ reduction during the COVID-19 outbreak in early 2020. This analysis may shed light on the parameterization of NO_2_ emission related to human mobility, as well as the understanding of the effect of transportation on atmospheric NO_2_. The indices based on the Baidu Big Data are able to provide daily information on human activities, and thus can predict the change of NO_2_. In future, the data could be used to modify the emission model and make the emission estimation more accurate [37,38].

In this study, we employed a simplified model from Ref. [27] to describe the dependence of atmospheric NO_2_ on emissions and meteorology. Compared with physical and chemical transfer models, which require a large source of computing time [26] and are affected by uncertainties in modelled physical or chemical processes, the approach shown in this work is fast and accurate with relative error <5% even in the heaviest polluted area in China, provided the emission does not change significantly. Our findings demonstrate the reliability of statistic approaches to predict tropospheric NO_2_ changes.

We suggest further efforts to develop novel statistic-based approaches as important supplements to the chemical transport models, particularly to understand the sensitivity of NO_2_ to various meteorological variables to provide more accurate predictions. The uncertainty in the statistic model deserves further studies, in particular its dependence on the background NO_2_ concentration, as well as the sensitivity of NO_2_ to each selected meteorological parameter. More meteorological parameters or more complicated functions to describe the dependence of atmospheric NO_2_ on meteorology conditions, also deserve further study.

## DATA AND METHODS

### Data

The standard product of tropospheric NO_2_ column density retrieved from the Ozone Monitoring Instrument (OMI) onboard Aura satellite (OMNO2, Level 2, version 003, available at https://disc.gsfc.nasa.gov/datasets/OMNO2_003/summary) was used in this study. Original orbit data were gridded into 0.25 * 0.25 degree to collocate with reanalysis data. To exclude the potential impacts from cloud contamination, only samples with cloud fraction <30% and NO_2_ column concentration <50 $\times $ 10^15^ molec/cm^2^ were used in our study. Row anomaly issues were carefully treated using the official quality flag of OMNO2 (see OMNO2 README file) based on the abnormal proportion of negative value in the data (if the ratio of negative value in an x-track was >2%, all the data in the track were not used).

The ECMWF atmospheric reanalysis data (ERA5, Single Level and Pressure Level) were used to provide meteorological parameters in this study. Original ERA5 data have a spatial resolution of 0.25 * 0.25 degree and a temporal resolution of 1 hour.

I-index, E-index and C-index data are maintained by Baidu, Inc. and are available at https://qianxi.baidu.com/. The information is derived from billions of location requests per day using the Baidu Map app, with permission to share from users. All of the proxies are not absolute numbers of travelers but proportional values.

### Statistical model of atmospheric NO_2_

A multiple variable linear regression model was developed to quantify tropospheric nitrogen dioxide (NO_2_) as a function of meteorological factors, which was inspired by the model described by de Foy and Schauer [27] and Seo* et al.* [31], using combined satellite observations and atmosphere reanalysis data. Based on our statistics on the satellite retrievals (refer to Fig. S17) and the studies [28,39], the values of atmospheric column NO_2_ are log-normally distributed. Therefore, we used log(NO_2_) for the multiple regression analysis so that we could scale to a normal distribution with zero mean and unit standard deviation. Contributions from emission source are assumed unchanged with time in this model. The tropospheric NO_2_ is considered to be a linear function of five key meteorological factors [25]: planetary boundary layer height (PBLH), solar radiation (SR), surface temperature (T), relative humidity (RH) and wind speed (WS). To ensure all variables have similar order of magnitude, a logarithm transformation was conducted on NO_2_ column density, PBLH and SR. As a result, the regression model can be expressed as follows.

(5) \begin{eqnarray*} \log\! \left( {{\rm{N}}{{\rm{O}}_2}} \right) &=& {{\it {b}}_0} + {b_1} \cdot \log\! \left( {PBLH} \right)\nonumber\\ && +\, {b_2} \cdot \log\! \left( {SR + 10} \right) + {b_3} \cdot T\nonumber\\ && +\, {b_4} \cdot RH + {b_5} \cdot WS.\end{eqnarray*}

The coefficients of *b*_0_ to *b*_5_ are regression coefficients determined with the Iterative Reweighted Least Squares (IRLS) fitting method [27]. We used 2018 and 2019 data from 45 days before to 65 days after the Chinese Spring Festival (Chinese New Year based on Lunar Calendar) as training data to build up the regression model, and used 2017 data to test the model and assess the modelling error. Then we extended the model with estimated error to predict the column NO_2_ density in 2020, assuming there were no changes of anthropogenic emission. For more details on development of the model, see the Supplementary data.

### GEOS-Chem model simulation

The GEOS-Chem chemical transport model (http://www.geos-chem.org, version 12-8-1) is driven by assimilated meteorological data of MERRA-2 with nested 0.5° × 0.625° horizontal resolution. The GEOS-Chem model includes fully coupled O_3_-NOx-VOC-halogen-aerosol chemistry. The chemical boundary conditions are updated every 3 hours from a global simulation with 4° × 5° resolution. The model has been used to investigate O_3_ changes in China in recent literature [40,41]. Emissions in GEOS-Chem are computed by the Harvard-NASA Emission Component (HEMCO). Global default anthropogenic emissions are from CEDS (Community Emissions Data system) [42]. Regional emissions are replaced by MEIC (Multiresolution Emission Inventory for China) in China, MIX in other regions of Asia [38]. The total anthropogenic NOx and VOCs emission in MEIC inventory are further scaled based on public literature [12,43] to obtain the annual emission in 2019. Open fire emissions are from the Global Fire Emission Database (GFED4) [44]. Natural emissions of O_3_ precursors, including NOx from lightning and soil and VOCs from vegetation are calculated on the basis of the assimilated MERRA-2 meteorology. The biogenic emissions of VOCs are calculated according to the Model of Emission of Gases and Aerosols from Nature (MEGAN v2.10) [45].

### DATA AVAILABILITY

NO_2_ data are available at https://disc.gsfc.nasa.gov/datasets/OMNO2_003/summary. The ERA5 meteorological data are from https://www.ecmwf.int/en/forecasts/datasets/reanalysis-datasets/era5. The migration data are from https://qianxi.baidu.com/.

### CODE AVAILABILITY

The computer codes used to analyze the data are available from the corresponding author on reasonable request.

## Supplementary Material

nwab061_Supplemental_Files
