# Supplementary material for: Untangling the contributions of meteorological conditions and human mobility to tropospheric NO2 in Chinese mainland during the COVID-19 pandemic in early 2020
Source: Natl Sci Rev. 2021 Apr 9;8(11):nwab061. doi: 10.1093/nsr/nwab061 (PMC8083328; doi:10.1093/nsr/nwab061)
Supplement: nwab061_Supplemental_Files [file nwab061_supplemental_files.zip › Supplementary Data File.pdf]

Supplement Information of

Untangle the contributions of meteorology condition and human mobility on tropospheric NO_2 in the mainland of China during COVID-19 pandemic in early 2020

Zhang Yuxiang[a,b], Bo Haixu[a,c], Jiang Zhe[a], Wang Yu[a], Fu Yunfei[a], Cao Bingwei[d], Wang Xuewen[e], Chen Jiaqi[a] and Li Rui[a,b,c*]

[a] *School of Earth and Space Sciences, University of Science and Technology of China, Hefei,230026, China*

[b] *Comparative Planetary Excellence Innovation Center, Chinese Academy of Sciences, Hefei,230026, China*

[c] *State Key Laboratory of Fire Science, University of Science and Technology of China, Hefei, 230026, China*

[d] *Jiangxi Ecological Environment Monitoring Center, Nanchang, China*

[e] *Green Earth Research Inc., Slingerlands, 12259, Newyork, USA*

[+] Zhang Yuxiang Bo Haixu and Jiang Zhe are co-first authors who made equal contributions to this study.

[*] Corresponding authors: rli7@ustc.edu.cn

List of Supplementary Figures

Figure S1. The Scatter Plot of model predicted NO_2 against satellite observed NO_2 at 0.5×0.5 degree with (upper row) and without (lower row) samples over 50×10^{15} molec/cm^2 in 2017 (a, b) both months, (c, d) Month-01, (e, f) Month-02.

Figure S2. Variations of spatial and temporal (daily) correlation coefficients between satellite observation and model prediction of NO_2 with increasing threshold of truncation of observed column NO_2 concentration. From left to right: 2017; 2020; 2018; 2019.

Figure S3. Spatial and temporal (daily) correlation coefficients between satellite observation and model prediction of NO_2 as function of the size of spatial unit (the grid) for regression: 0.5×0.5 degree; 1.0×1.0 degree; 2.0×2.0 degree; and 3.0×3.0 degree. From left to right: 2017; 2020; 2018; 2019.

Figure S4. The observed NO_2 (OBS), modelled NO_2 (MOD) and difference (MOD-OBS) in eight periods (one month before and after Spring Festival in recent four years).

Figure S5. The temporal correlation between daily OBS and MOD in 34 provinces. Only the provinces with correlations passed 95% significance test are filled with colors.

Figure S6. The temporal correlation between daily OBS and MOD in 343 cities. Only the cities with correlations coefficients passed 95% significance test are filled with colors.

Figure S7. The time series of daily satellite observed NO_2 (blue line), model predicted NO_2 (red line), and the difference between them (orange bar: model>observation, green bar: model<observation) in 34 provinces in the mainland of China in 2017. The vertical black dash line denotes Spring Festival. The figures are sorted according to the average NO_2 column amount in 2017 (high to low).

Figure S8. Same as **Figure S7**, but for 2018.

Figure S9. Same as **Figure S7**, but for 2019.

Figure S10. Same as **Figure S7**, but for 2020.

Figure S11. The isolated contribution of emission and meteorology to the changes of NO_2 between 2020 and 2018 in 172 China's cities with monthly mean NO_2 over 3×10^{15}molec/cm^2. (a) Relative reduction of satellite observed atmospheric NO_2 (%) (b) Estimated emission induced reduction of NO2 (%); (c) Estimated weather induced changes of NO_2 (%); (d) Modelling error of the estimation (%).

Figure S12. The isolated contribution of emission and meteorology to the changes of NO_2 between 2020 and 2019 in 155 China's cities with monthly mean NO_2 over 3×10^{15}molec/cm^2. (a) Relative reduction of satellite observed atmospheric NO_2 (%) (b) Estimated emission induced reduction of NO2 (%); (c) Estimated weather induced changes of NO_2 (%); (d) Modelling error of the estimation (%).

Figure S13. The averaged immigration index (I-index), emigration index (E-index) and intra-city index (C-index) one month before and after Spring Festival in 2019 and 2020.

Figure S14. The time series of logrNO_2, mobility indices for 2019 in different provinces. The figures are listed according to the average NO_2 column amount in 2017 (high to low). Yellow bar: positive logrNO_2, green bar: negative logrNO_2, red line: immigration index (I-index), red dash line: emigration index (E-index), blue line: intra-city activity intensity index (C-index), vertical black line: Spring Festival.

Figure S15. Same as **Figure S14**, but for 2020.

Figure S16. The comparison of PBLH, Relative Humidity, Solar Radiation, Temperature and Wind Speed in 2017 (upper row), 2020 (middle row) and the associated difference (lower row, masked with monthly mean NO_2 over 3×10^{15}molec/cm^2).

Figure S17. The relationships between NOx emission reduction and the changes of atmospheric column NO_2 under different levels of VOCs emission reduction in China in 2020 Feb as derived from the GEOS-Chem chemical transport model simulation with unchanged meteorology conditions.

Figure S18. The probability distribution function of of satellite (Aura/OMI) observed atmospheric NO_2 (a) number concentration and (b) the associated value in logrithm, i.e. Log(NO_2)

The modelling of tropospheric NO_2 as function of meteorological variables

Establishment of the model

A multiple variables linear regression model was developed to quantify the column density of tropospheric NO_2 as function of meteorological factors [1-3]. This is similar to the model described by *de Foy and Schauer* (2015) [1] and *Seo J.* et. al. (2018) [4]. In this model, tropospheric NO_2 is considered as a linear function of five key meteorological factors of planetary boundary layer height (PBLH), surface solar radiation (SR), surface temperature (T), relative humidity (RH) and wind speed (WS) [1, 5-8]. To make sure all of the values of independent variables and function have a similar order of magnitude, a logarithm transformation is conducted on NO_2, PBLH and SR. Meanwhile, negative and zero values in NO_2 data are deleted and an offset is given to SR. As a result, the regression model can be expressed as follows.

$$\log(\mathrm{NO_2}) = b_0 + b_1 \cdot \log(PBLH) + b_2 \cdot \log(SR + 10) + b_3 \cdot T + b_4 \cdot RH + b_5 \cdot WS$$

The coefficients of b_0 to b_5 are regression coefficients determined by the least square fitting method. As least square procedure is sensitive to outliers, NO_2 value higher than 50×10^{15} molec/cm^2 was excluded (~0.1% of total samples).

We used Iterative Reweighted Least Squares (IRLS) [1-3, 6, 8] to improve the robustness of the regression. After one step of individual least square regression, residual errors of all the data points are checked and compared with the standard error (STD). Only the data points with residual error less than 3 times of the STD was retained and used in the next round of least square regression. This loop will go on until all the residual errors of the remaining data are within 3 times of STD.

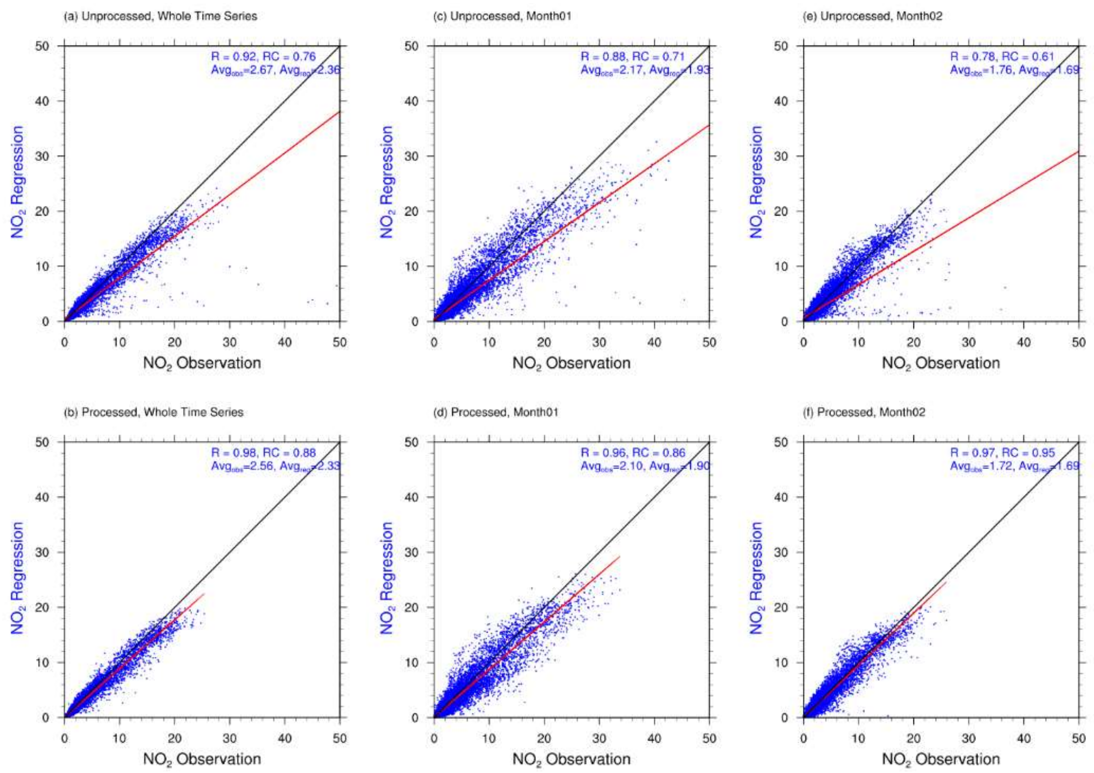

Figure S1. The Scatter Plot of model predicted NO_2 against satellite observed NO_2 at 0.5×0.5 degree with (upper row) and without (lower row) samples over 50×10^{15} molec/cm^2 in 2017 (a, b) both months, (c, d) Month-01, (e, f) Month-02.

The quantitative relationships between NO_2 and meteorology conditions are expected varying with topography, land cover, local climate regime, and more important the local emission intensity [9]. Therefore, we conducted the regression in each grid of 0.5×0.5 ° (i.e. the regression spatial unit), in which the satellite observed NO_2 and ERA5 reanalyzed meteorology parameters were collocated. We used data in 2018 and 2019 from 45 days before to 65 days after the Chinese Spring Festival (**Table S1**) as training data to build up the regression model and used data in 2017 during the same period to test the model and assess the modelling error. Then we applied the model to predict the column NO_2 density in year 2020 as function of associated meteorology conditions assuming no changes of anthropogenic emission. It should be noticed the date of 2020 Spring Festival is the closest to that in 2017, therefore we choose 2017 as the reference years for discussing the reduction of NO_2 in the manuscript.

Table S1. The dates of Spring Festival in recent 4 years

Year	Spring Festival
2017	2017.01.28
2018	2018.02.16
2019	2019.02.05
2020	2020.01.25

Sensitivity tests were conducted to determine the optimal values of the threshold of abnormal

NO_2 value (50×10^{15} molec/cm^2), the size of the regression spatial unit (i.e. 0.5×0.5 degree), the data length (i.e. 110 days) and the start date and end date for regression (i.e. 45 days before to 65 days after the Chinese Spring Festival).

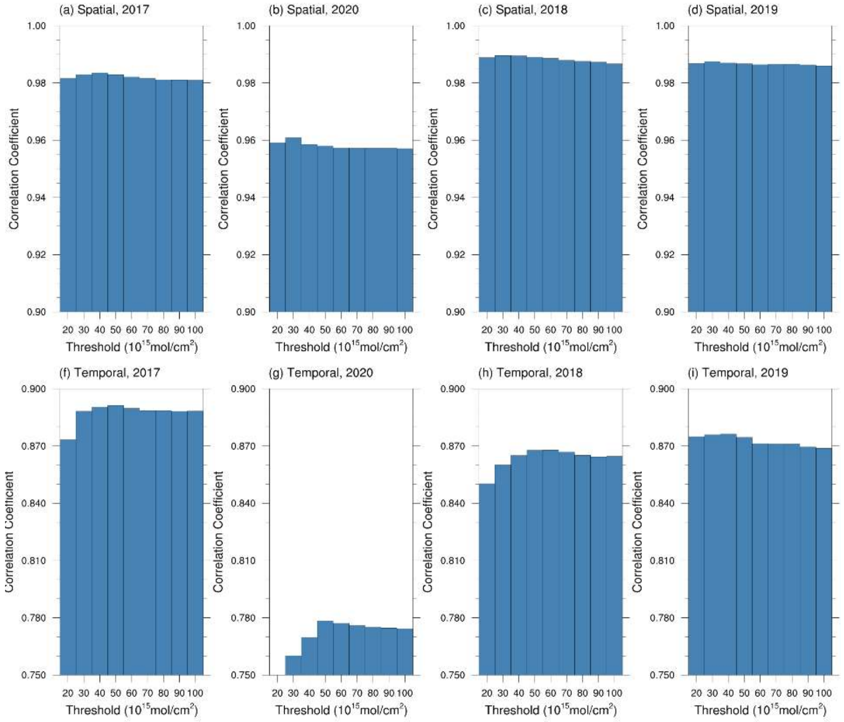

Figure S2. Variations of spatial and temporal (daily) correlation coefficients between satellite observation and model prediction of NO_2 with increasing threshold of truncation of observed column NO_2 concentration. From left to right: 2017; 2020; 2018; 2019

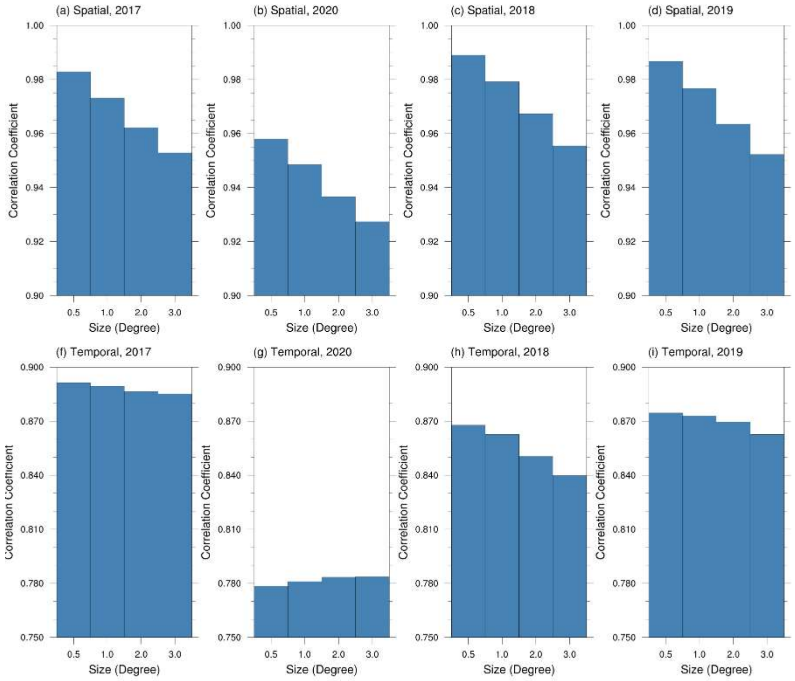

Figure S3. Spatial and temporal (daily) correlation coefficients between satellite observation and model prediction of NO_2 as function of the size of spatial unit (the grid) for regression: 0.5×0.5 degree; 1.0×1.0 degree; 2.0×2.0 degree; and 3.0×3.0 degree. From left to right: 2017; 2020; 2018; 2019.

Validation of the model at monthly mean scale

The model predicted monthly mean NO_2 in Month-01 (one month before Spring Festival) and Month-02 (one month after Spring Festival) from 2017 to 2020 are compared to the associated OMI observations (**Figure S4**).

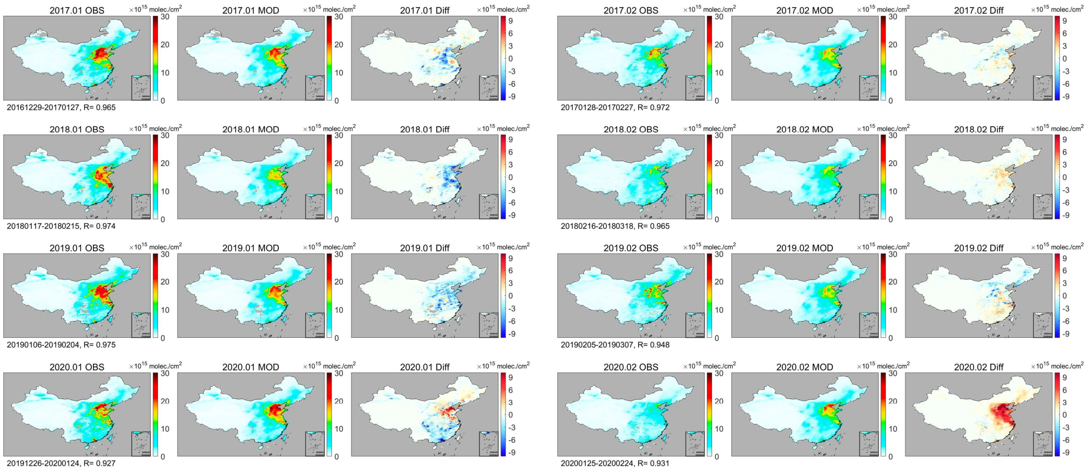

Figure S4. The observed NO_2 (OBS), modelled NO_2 (MOD) and difference (MOD-OBS) in eight periods (one month before and after Spring Festival in recent four years).

As a self-consistence check in 2018 and 2019, the model prediction of NO_2 matches the satellite observations very well with spatial correlation coefficients 0.95-0.98 ($p<0.001$). The model shows a slight "negative" bias in Month-01 and a slight "positive" bias in Month-02. However, both of the absolute value of such bias are almost negligible in the wide western China. Even in the eastern and central China with the highest NO_2 concentration of 20-30×10^{15} molec/cm^2, the model prediction bias is less than 3×10^{15} molec/cm^2 (<~10%).

More importantly, the performance of the model prediction is even better in 2017 with satellite and meteorology data all independent from the model's training data. The spatial correlation coefficients between model and satellite NO_2 are 0.965-0.973 ($p<0.001$). The bias of the model prediction is negligible in most area in China in Month-02 2017. In the heaviest polluted area of central and eastern China, the bias is typically less than 1×10^{15} molec/cm^2 (3-5%). This is a strong evidence that the model can be applied to adjacent years to predict the atmospheric NO_2 if there is no significant change of emission.

In Month-01 2020, the bias of model prediction was significantly larger than that in other years. In Month-02 2020, the model prediction turned to be about twice higher than the real satellite observations indicating the basic hypothesis of the model [10], i.e. no changes of emission,

completely failed.

Validation of the model at daily scale

To exam the consistence of the model prediction with satellite observations at daily scale, we calculated the temporal correlation coefficients at 34 provinces (**Figure S5**) and 343 cities (**Figure S6**) in the mainland of China.

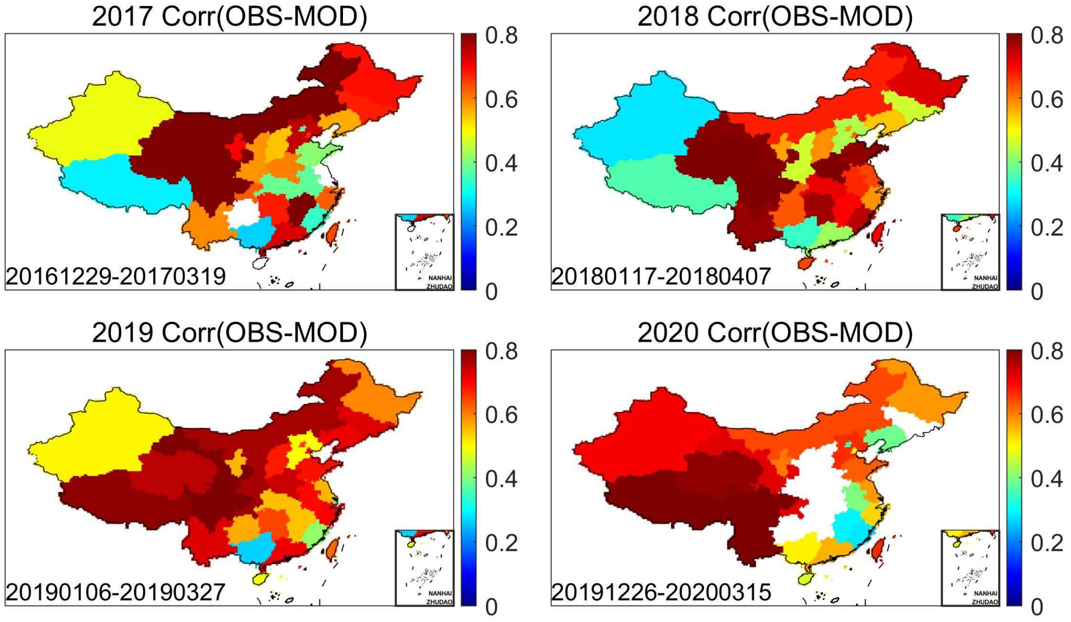

Figure S5. The temporal correlation between daily OBS and MOD in 34 provinces. Only the provinces with correlations passed 95% significance test are filled with colors.

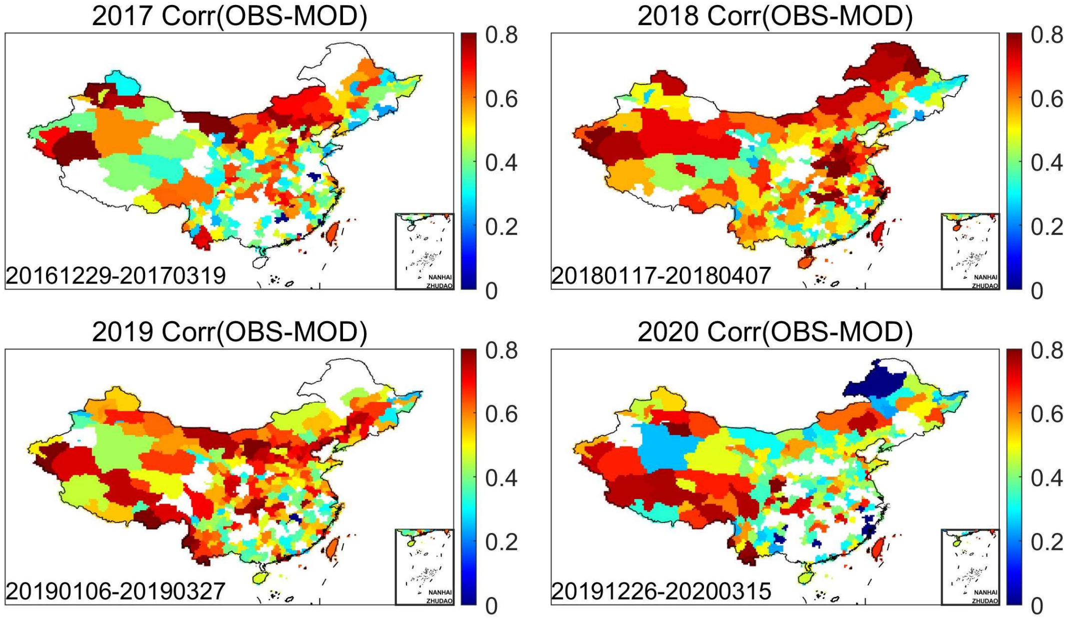

Figure S6. The temporal correlation between daily OBS and MOD in 343 cities. Only the cities with correlations coefficients passed 95% significance test are filled with colors.

Even at daily level, the statistical based model still can capture the temporal variations of real atmospheric NO_2 very well in all provinces in 2018 and 2019, and in most provinces in 2017 except Jiangsu and Guizhou (**Figure S5**). At smaller spatial scale of cities, the model prediction still can represent the temporal variation of NO_2 in most cities in 2017, 2018 and 2019 (**Figure S6**). In 2020, the temporal correlations of daily NO_2 between model and satellite observation are still positive significantly in a lot of provinces except those in central and southern China (**Figure S5**). And also, more cities in Central China show insignificant temporal correlations due to the dramatic change of emissions (**Figure S6**).

The time series of daily NO_2 at 34 provinces in 2017-2020 are shown in **Figure S7-S10** for references. Generally, in those provinces with relatively heavy pollution, the modelling results in 2017-2019 captured the observations well, and the positive (orange bars) and negative (green bars) modelling error are randomly distributed. However, in 2020, most of the modelling error after Spring Festival are dominated by positive value (orange bars) indicating large overestimation of the model prediction [10, 11].

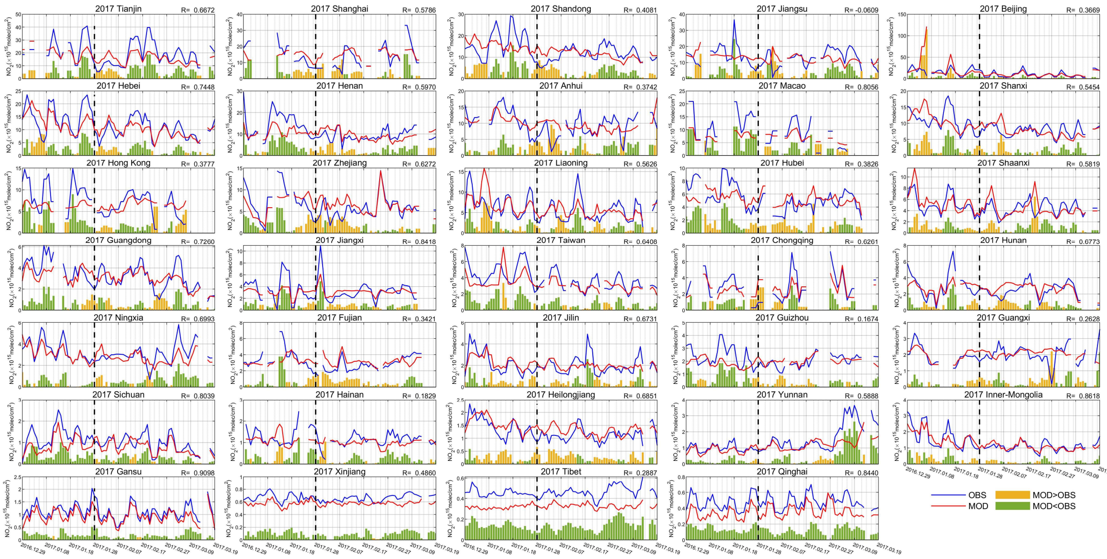

Figure S7. The time series of daily satellite observed NO_2 (blue line), model predicted NO_2 (red line), and the difference between them (orange bar: model>observation, green bar: model<observation) in 34 provinces in the mainland of China in 2017. The vertical black dash line denotes Spring Festival. The figures are sorted according to the average NO_2 column amount in 2017 (high to low).

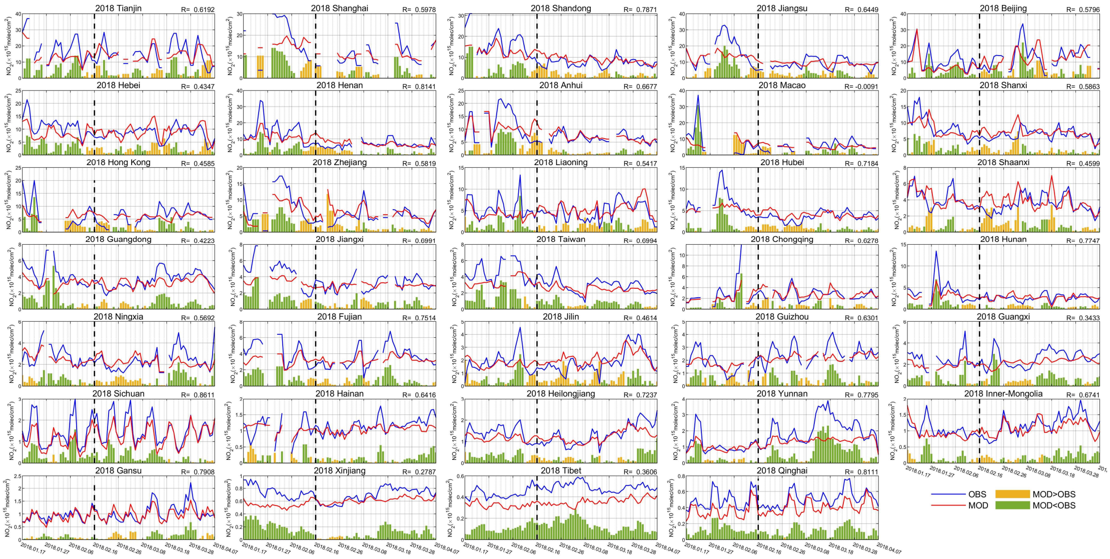

Figure S8. Same as **Figure S7**, but for 2018.

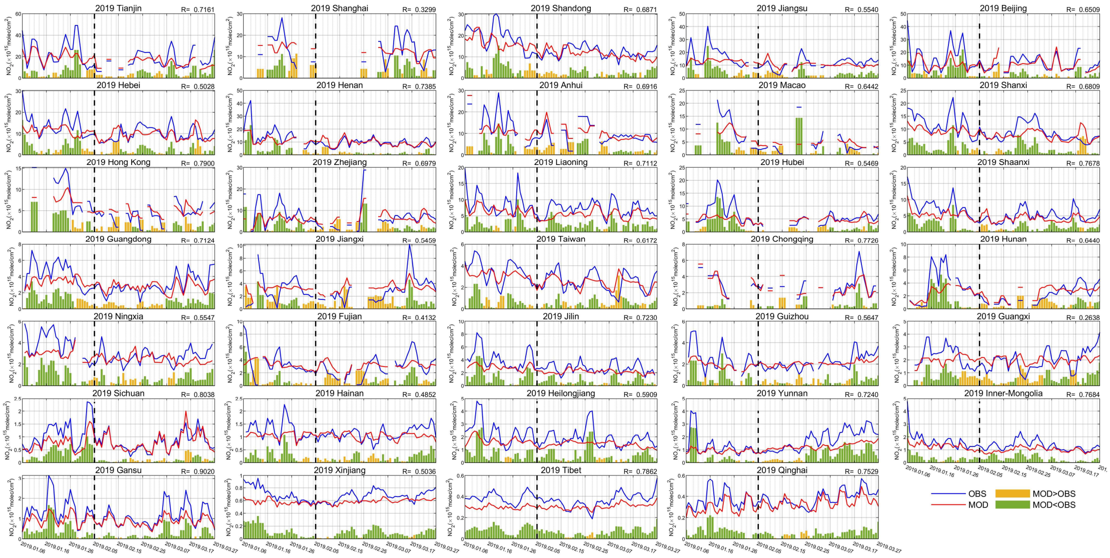

Figure S9. Same as **Figure S7**, but for 2019.

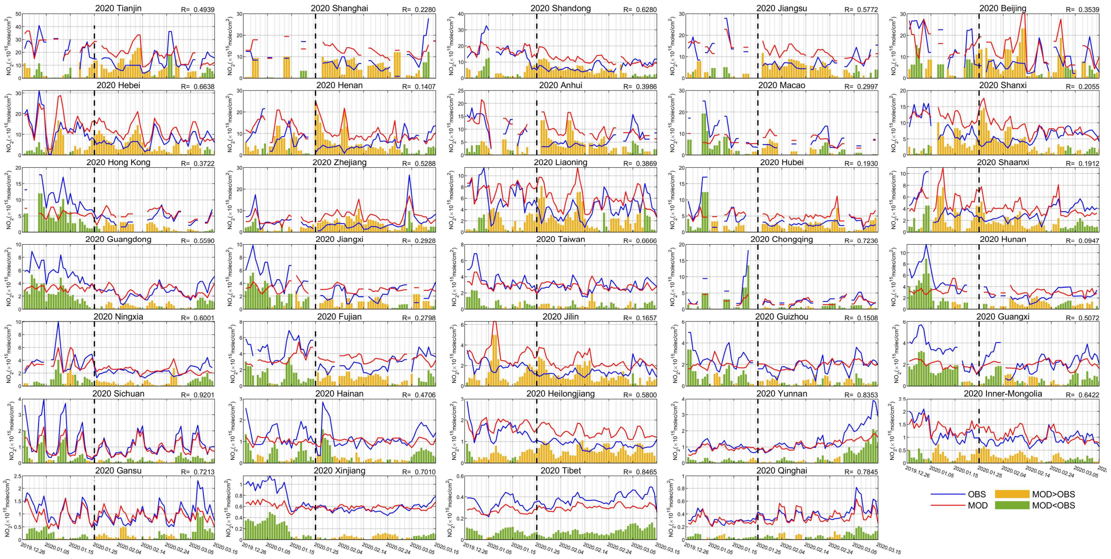

Figure S10. Same as **Figure S7**, but for 2020.

Relative contributions from emission, meteorology to satellite observed reduction of NO_2 in 2020

As discussed in the manuscript, emission induced reduction of NO_2 in 2020 comparing to 2017 actually is greater than we saw from satellite observations. This is because the weather effect in 2020 actually led to an increase of NO_2. Similar analysis was conducted using 2018 and 2019 as reference years. The results are shown in **Figure S11-12**.

Comparing to 2018, the emission induced reduction of NO_2 in 2020 is very high, at -62.5$\pm$31.4%. The satellite observed difference of NO_2 between 2020 and 2018 is just -26.5$\pm$18.1%. The meteorology effect contributes an increase of 24.4$\pm$21.5%. This is partially because the date of Spring Festival of 2018 is at Feb 16, which is 21 days later than that in 2020. The weather in 2018 Month-02 was warmer and more instable than that in 2020 Month-02. And the modelling error shown in **Figure S10** also is relatively large at -11.6$\pm$18.4%.

Comparing to 2019, the emission induced reduction of NO_2 in 2020 is -47.9$\pm$22.9%. The satellite observed difference of NO_2 between 2020 and 2019 is -44.3$\pm$18.8%. The meteorology effect contributes an increase of 10.1$\pm$15.0%. And the modelling error shown in **Figure S11** is 6.6$\pm$18.4%. These results are close to the comparison between 2020 and 2017 as discussed in the manuscript.

Overall, when comparing 2020 to 2018 and 2019, the emission induced reduction of NO_2 all are significantly larger than the satellite observed reduction due to the meteorology contribution. This confirmed the conclusion in our manuscript.

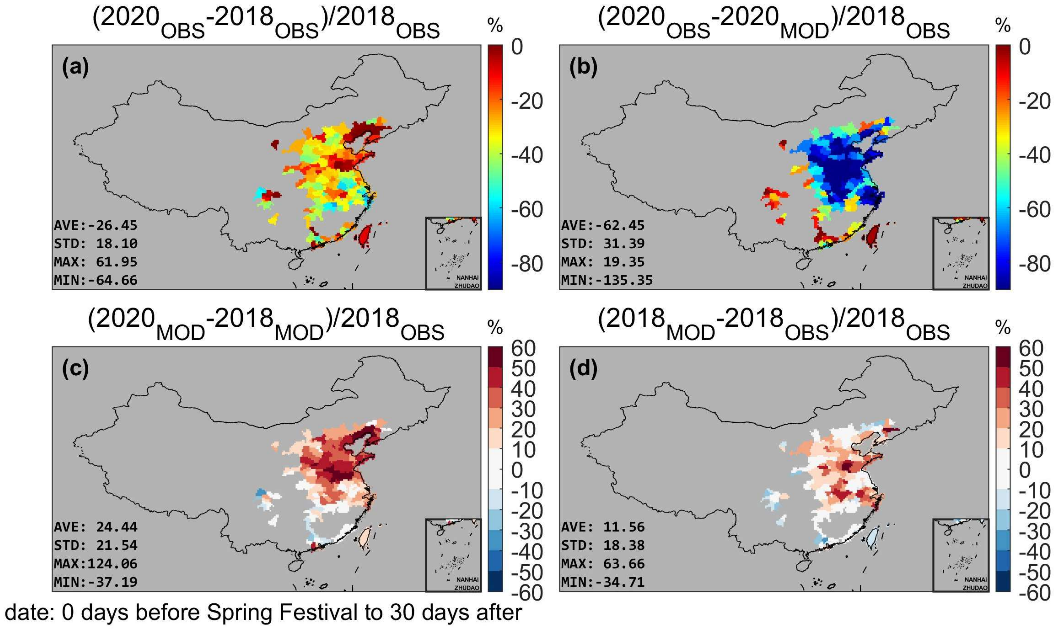

Figure S11. The isolated contribution of emission and meteorology to the changes of NO_2 between 2020 and 2018 in 172 China's cities with monthly mean NO_2 over 3×10^{15} molec/cm^2. (a) Relative reduction of satellite observed atmospheric NO_2 (%) (b) Estimated emission induced reduction of NO_2 (%); (c) Estimated weather induced changes of NO_2 (%); (d) Modelling error of the estimation (%).

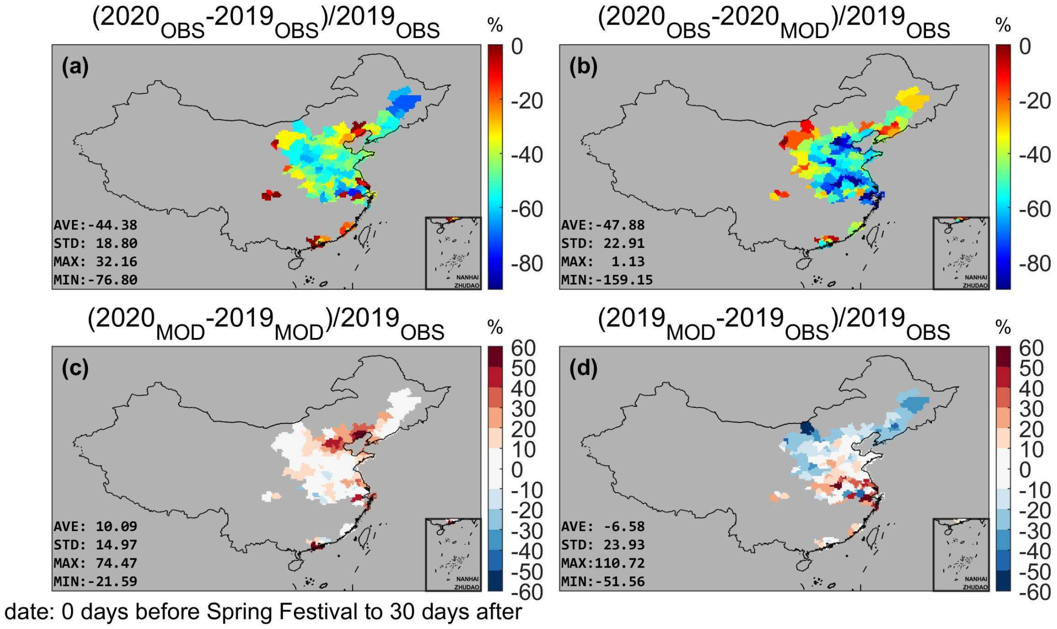

Figure S12. The isolated contribution of emission and meteorology to the changes of NO_2 between 2020 and 2019 in 155 China's cities with monthly mean NO_2 over 3×10^{15} molec/cm^2. (a) Relative reduction of satellite observed atmospheric NO_2 (%) (b) Estimated emission induced reduction of NO_2 (%); (c) Estimated weather induced changes of NO_2 (%); (d) Modelling error of the estimation (%).

Spatial pattern of Baidu's human mobility indices

Baidu Inc. provided daily traveling data of the relative number of travel population between cities and inside cities at http://qianxi.baidu.com. The indices are not absolute numbers, but proxies to present the intensity [12-14]. There are several kinds of indices provided by Baidu. In our study, we use three indices, immigration index (I-index), emigration index (E-index), and intra-city index (C-index). I-index in each city presents the population moving flow into the city, E-index presents that out of the city, and C-index indicates the proportion of the people moving inside the city to the total population of the city.

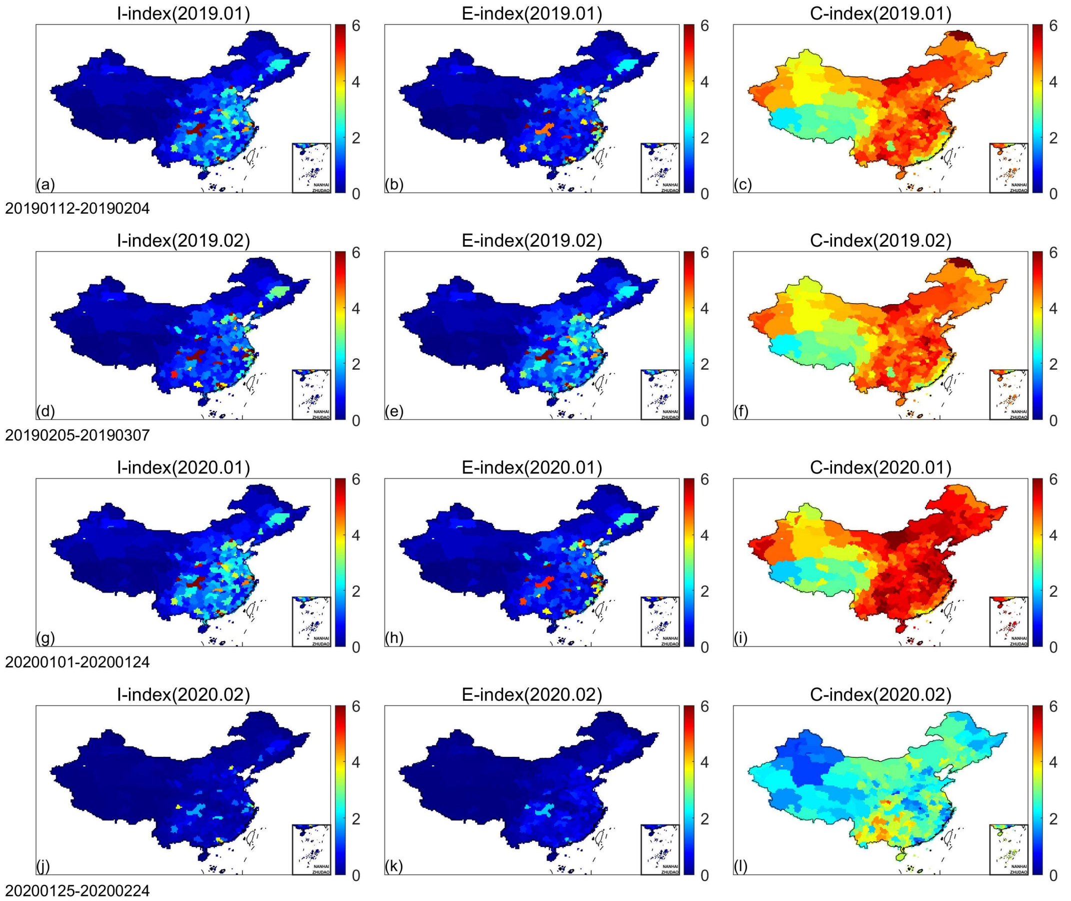

Figure S13. The average immigration index (I-index), emigration index (E-index) and intra-city index (C-index) one month before and after Spring Festival in 2019 and 2020.

Figure S13 shows the three original Baidu immigration indices averaged over one month before and after Spring Festival in 2019 and 2020. Generally, the values are higher in the eastern part of China than those in western part of China. The spatial pattern and inhomogeneities are associated with population density and activities in economy. Those megacities such as Beijing, Shanghai, Guangzhou, and Chongqing etc. show large values of all indices because of large number of foreign workers and prosperous economy. The Spring Festival effect is shown in **Figure S13**. For

example, the distribution of moving out index E-index in 2019 Month-01 is similar to the moving in index I-index in 2019 Month-02, this is associated with the population flow of family reunion for Spring Festival and the flow of coming back for work after.

The indices clearly show the effect of national wide response to COVID-19 started from Jan 23 2020, two days before the Spring Festival. In Month-01 2020, I-index and E-index show similar distribution and intensity as Month-01 2019, and C-index was even higher in most cities, which indicate travelers were not aware of the outbreak of COVID-19 and didn't change their travel plan. However, in Month-02 2020, I-index and E-index dropped much lower in most cities, and C-index reduced over a half, especially in Hubei because of the strict self-quarantine required by the government [13].

Temporal Variations of daily human mobility indices and emission-induced reduction of NO_2

The time series of daily human mobility indices and emission-induced reduction of NO_2 in megacities with populations over 8 million are shown in the manuscript **Figure 3**. Similar results at provinces (34 in total) in the mainland of China are shown in **Figure S14** and **Figure S15** for 2019 and 2020. In 2019 (**Figure S14**), the emission induced change of NO_2, i.e. logrNO_2, is generally small in 2019, most of the values are within the range of standard deviation, and its sign (positive or negative) is relatively random. In 2020 (**Figure S15**), all three migration indices decrease a lot after 22nd Jan 2020, and start to increase in the late Feb except Hubei, showing the measures for COVID-19 event in China [13, 15]. The emission induced reduction of logrNO_2 in 2020 are constantly and significantly positive in more than half of provinces after Jan 23rd 2020, the start of lockdowns. This is evident particularly in those heavily polluted areas such as Tianjin, Shanghai, Shandong, Jiangsu etc. indicating the strong impacts from the quarantine [16].

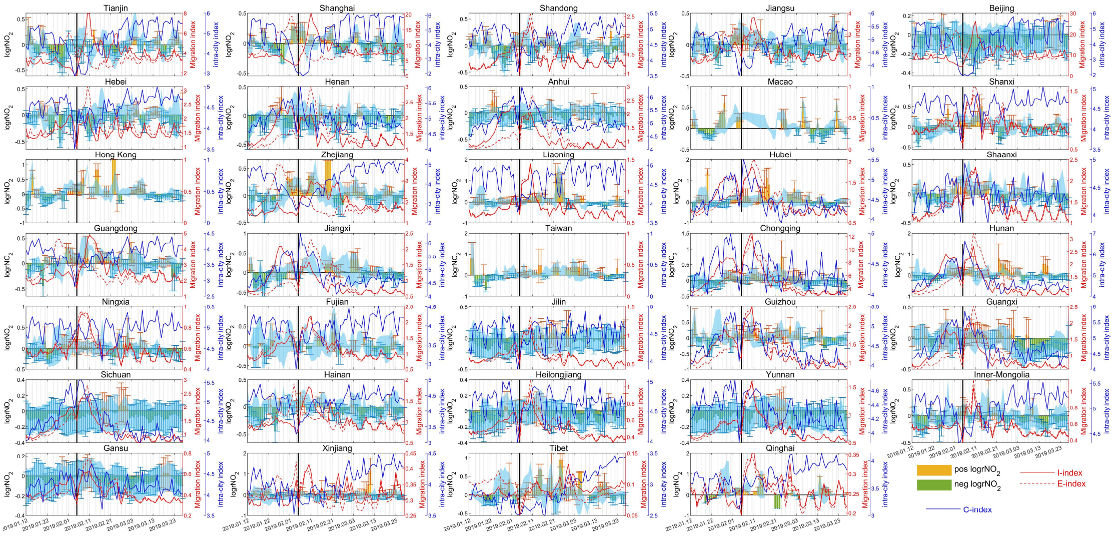

Figure S14. The time series of logrNO_2, mobility indices for 2019 in different provinces. The figures are listed according to the average NO_2 column amount in 2017 (high to low). Yellow bar: positive logrNO_2, green bar: negative logrNO_2, red line: immigration index (I-index), red dash line: emigration index (E-index), blue line: intra-city activity intensity index (C-index), vertical black line: Spring Festival.

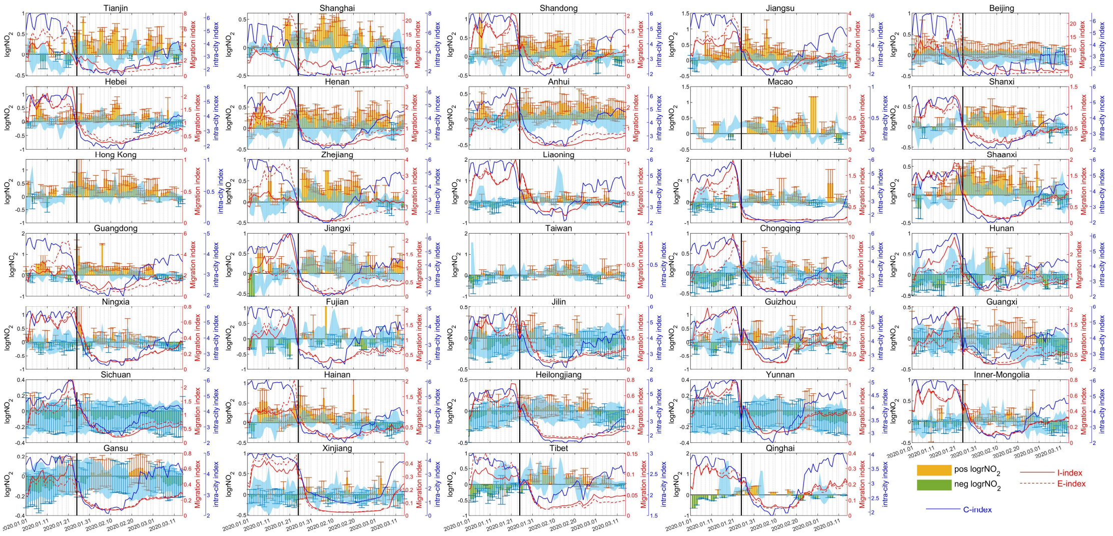

Figure S15. Same as **Figure S14**, but for 2020.

The reduction of anthropogenic emission (Figure 2b) was the strongest in Beijing-Tianjin-Hebei area, Shangdong peninsula, Yangtze River Delta area, but was relatively weak in Liaoning province, Inner Mongolia, and etc. With the strict prohibition of human activity during the 2020 COVID-19 pandemic, the reduction of anthropogenic emission in economically developed area was very significant, and thus induced strong impacts on the atmospheric NO_2. While in those economically underdeveloped area with low population density, such effect was weak. In Taiwan, where no quarantine was implemented at that time, the emission-induced effect was close to zero.

On the other hand, the changes of meteorology condition were different in our study area.

As shown in this **Figure S16**, the PBLH and solar radiation in 2020 are both lower than in 2017 in eastern areas especially around Beijing-Tianjin-Hebei area. Meanwhile, the relative humidity in 2020 is higher in those areas than in 2017. And the difference of temperature between 2020 and 2017 is small. In the north of Beijing-Tianjin-Hebei area, wind speed is lower in 2020, while in the south, wind speed shows higher value in 2020. In some areas in middle China, e.g. Anhui, wind speed of 2020 is slower than in 2017. Consequently, in Beijing-Tianjin-Hebei area, the lower radiation, higher relative humidity, lower PBLH and weaker wind speed lead to remarkably positive effects on atmospheric NO_2. While in the southern polluted areas, the impacts from multiple meteorology parameters on NO2 somewhat cancel out each other and lead to neutral effects with relatively large uncertainties.

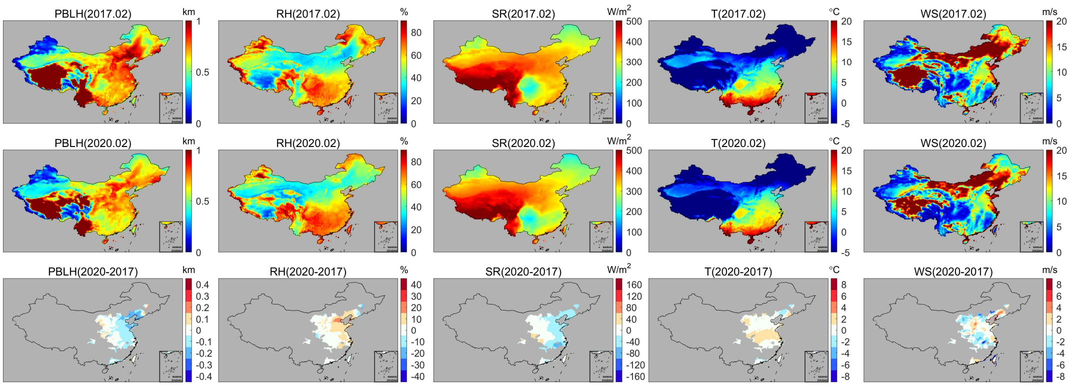

Figure S16. The comparison of PBLH, Relative Humidity, Solar Radiation, Temperature and Wind Speed in 2017 (upper row), 2020 (middle row) and the associated difference (lower row, masked with monthly mean NO_2 over 3×10^{15}molec/cm^2).

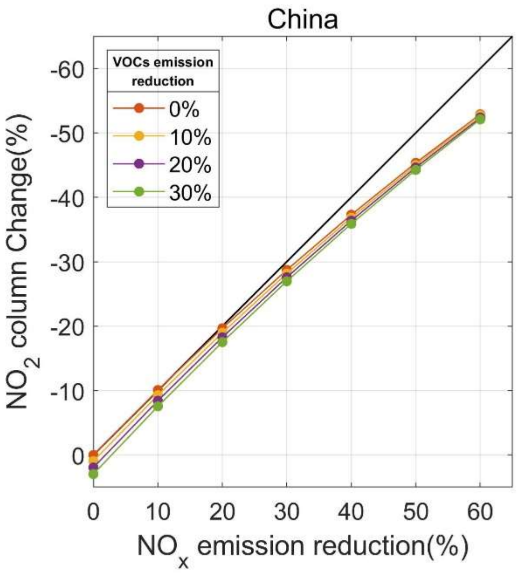

Figure S17. The relationships between NOx emission reduction and the changes of atmospheric column NO_2 under different levels of VOCs emission reduction in China in 2020 Feb as derived from the GEOS-Chem chemical transport model simulation with unchanged meteorology conditions.

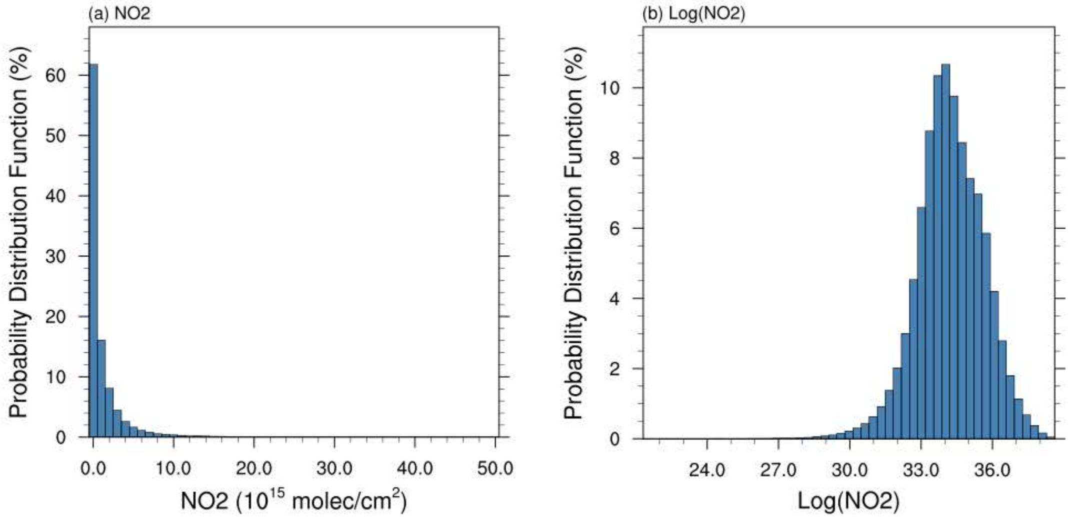

Figure S18. The probability distribution function of of satellite (Aura/OMI) observed atmospheric NO_2 (a) number concentration and (b) the associated value in logrithm, i.e. Log(NO_2)

Table S2. The coverage (%) of the areas with temporal correlation (logrNO_2 and indices, see **Figure 4**) passed 95% significance test in the provinces with high averaged NO2 concentration, and the mean explanations (R^2) of indices in each province.

Province	COV(I)	R^2 (I)	COV (E)	R^2 (E)	COV (C)	R^2 (C)
Jiangsu	85.7	0.347±0.103	82.1	0.267±0.112	100	0.493±0.160
Shandong	82.4	0.146±0.084	67.1	0.221±0.120	94	0.405±0.110
Henan	72.8	0.209±0.115	89	0.295±0.140	89	0.374±0.200
Tianjin	100	0.198±0.000	100	0.093±0.000	100	0.331±0.000
Zhejiang	86.4	0.208±0.073	59.2	0.232±0.052	100	0.308±0.128
Anhui	40.6	0.199±0.048	30	0.288±0.129	87.4	0.279±0.125
Beijing	0	NaN	100	0.080±0.000	100	0.248±0.000
Hebei	48.1	0.107±0.021	43.2	0.129±0.042	58.5	0.237±0.056
Shanxi	7.9	0.199±0.056	48.5	0.115±0.037	54.4	0.186±0.095
Shaanxi	16.2	0.228±0.036	16.2	0.240±0.081	71.2	0.172±0.110
Shanghai	0	NaN	100	0.120±0.000	100	0.142±0.000
Hubei	43.1	0.137±0.036	35.7	0.132±0.036	35.7	0.132±0.030
Liaoning	20.3	0.074±0.018	25.9	0.104±0.030	42.3	0.089±0.055

Generally, in most of the presented provinces, it can be seen that the coverages of C-index are larger than those of I-index and E-index. The coverages in those polluted provinces such as Shandong, Jiangsu, Henan, are high (about 90%). Also, the explanations of C-index are larger than I-index and E-index, except in Hubei and Shaanxi. In Hubei, the explanation of I-index is 0.137, that of E-index is 0.132, and that of C-index is 0.132, slightly lower than that of I-index. And in Shaanxi, the explanation of I-index is 0.228, that of E-index is 0.240, and that of C-index is 0.172.

In other places, such as Shandong, Jiangsu, Henan, C-index can explain about 40% of the temporal change of NO_2. So we believe that in most areas, C-index is better performed to predict the temporal change of NO_2.

Reference

1. de Foy, B, Schauer, JJ. Origin of high particle number concentrations reaching the St. Louis, Midwest Supersite. *Journal of Environmental Sciences*. 2015; **34**: 219-31.
2. de Foy, B, Lu, Z, Streets, DG. Satellite NO2 retrievals suggest China has exceeded its NOx reduction goals from the twelfth Five-Year Plan. *Scientific Reports*. 2016; **6**(1): 35912.
3. de Foy, B, Lu, Z, Streets, DG. Impacts of control strategies, the Great Recession and weekday variations on NO2 columns above North American cities. *Atmospheric Environment*. 2016; **138**: 74-86.
4. Seo, J, Park, DSR, Kim, JY*, et al.* Effects of meteorology and emissions on urban air quality: a quantitative statistical approach to long-term records (1999–2016) in Seoul, South Korea. *Atmos Chem Phys*. 2018; **18**(21): 16121-37.
5. Wang, L, Wang, J, Tan, X*, et al.* Analysis of NOx Pollution Characteristics in the Atmospheric Environment in Changchun City. *Atmosphere*. 2020; **11**(1).
6. de Foy, B. City-level variations in NOx emissions derived from hourly monitoring data in Chicago. *Atmospheric Environment*. 2018; **176**: 128-39.
7. Le, TH, Thanh Nguyen, TN, Lasko, K*, et al.* Vegetation fires and air pollution in Vietnam. *Environmental Pollution*. 2014; **195**: 267-75.
8. de Foy, B, Schauer, J, Helmig, D*, et al.* Changes in speciated PM2.5 concentrations in Fresno, California, due to NOx reductions and variations in diurnal emission profiles by day of week. *Elem Sci Anth*. 2019; **7**.
9. Zheng, B, Tong, D, Li, M*, et al.* Trends in China's anthropogenic emissions since 2010 as the consequence of clean air actions. *Atmos Chem Phys*. 2018; **18**(19): 14095-111.
10. Bauwens, M, Compernolle, S, Stavrakou, T*, et al.* Impact of coronavirus outbreak on NO2 pollution assessed using TROPOMI and OMI observations. *Geophysical Research Letters*. 2020; **n/a**(n/a): e2020GL087978.
11. Shi, X, Brasseur, GP. The Response in Air Quality to the Reduction of Chinese Economic Activities during the COVID-19 Outbreak. *Geophysical Research Letters*. 2020; **n/a**(n/a): e2020GL088070.
12. Chen, Z-L, Zhang, Q, Lu, Y*, et al.* Distribution of the COVID-19 epidemic and correlation with population emigration from Wuhan, China. *Chinese Medical Journal*. 2020; **133**(9).
13. Chen, H, Chen, Y, Lian, Z*, et al.* Correlation between the migration scale index and the number of new confirmed coronavirus disease 2019 cases in China. *Epidemiology and Infection*. 2020; **148**: e99.
14. Tian, H, Liu, Y, Li, Y*, et al.* An investigation of transmission control measures during the first 50

days of the COVID-19 epidemic in China. *Science*. 2020: eabb6105.
15. Chinazzi, M, Davis, JT, Ajelli, M*, et al.* The effect of travel restrictions on the spread of the 2019 novel coronavirus (COVID-19) outbreak. *Science*. 2020; **368**(6489): 395.
16. Wang, Q, Su, M. A preliminary assessment of the impact of COVID-19 on environment – A case study of China. *Science of The Total Environment*. 2020; **728**: 138915.
